# Dose- and Time-Dependent Modulation of Cx43 and Cx45 Expression and Gap Junction Conductance by Resveratrol

**DOI:** 10.3390/antiox15010088

**Published:** 2026-01-09

**Authors:** Gintarė Jančiukė, Rokas Mickus, Vytautas Raškevičius, Vytenis Arvydas Skeberdis, Ieva Sarapinienė

**Affiliations:** Institute of Cardiology, Lithuanian University of Health Sciences, 50162 Kaunas, Lithuania

**Keywords:** resveratrol, connexin 43, connexin 45, gap junction intercellular communication

## Abstract

Plant extracts are rich in various bioactive compounds, such as polyphenols, flavonoids, tannins, terpenoids, phenolic acids, saponins, alkaloids, and polysaccharides. Antioxidant polyphenols are increasingly attracting attention, not only as dietary components but also as valuable food industry byproducts. Resveratrol, present in a wide range of plants, is well recognized for its diverse biological activities, including antioxidant, antitumor, cardioprotective, and neuroprotective effects. Given the importance of intercellular communication in these physiological processes, gap junctions (GJs) composed of connexin (Cx) family proteins are of particular interest because they provide a direct pathway for electrical and metabolic signaling and are key players in maintaining normal organ function and cell development. Aberrations of GJ intercellular communication (GJIC) may result in the progression of cardiovascular and neurological diseases and tumorigenesis. Cx43 and Cx45 play crucial roles in cardiac excitation and contraction, and alterations in their expression are associated with disrupted impulse propagation and the development of arrhythmias. In this study, for the first time, we performed a comparative analysis of the effect of resveratrol on Cx43 and Cx45 GJIC using molecular modeling, a dual whole-cell patch-clamp technique to directly measure GJ conductance (g_j_), and other approaches. Our results revealed that resveratrol accomplished the following: (1) inhibited GJ g_j_ in Cx43- but enhanced it in Cx45-expressing HeLa cells; (2) exerted dose- and time-dependent changes in Cx expression and plaque size; (3) reduced cell viability and proliferation; (4) and altered Cx43 phosphorylation patterns linked to gating and plaque stability. Overall, resveratrol modulates GJIC in a dose-, time-, and connexin type-specific manner.

## 1. Introduction

Connexins (Cxs) are a large family of transmembrane proteins with four membrane-spanning domains, two extracellular loops, one intracellular loop, and intracellular N- and C-termini (CT) [1]. Cxs are synthesized in the endoplasmic reticulum, oligomerized in the Golgi, and delivered to the sarcolemma as hemichannels composed of six Cx subunits [2]. When hemichannels from adjacent cells align, they form GJs that enable electrical communication and the exchange of metabolites and signaling molecules [3]. Dense clusters of hundreds to thousands of individual channels form highly organized structures known as GJ plaques [4]. GJ conductance and permeability are modulated by factors such as junctional voltage, intracellular pH, divalent cations (Ca^2+^, Mg^2+^), phosphorylation, and various chemical agents [5,6]. Cxs interacting with cytoskeletal proteins, scaffolding molecules, and enzymes function not only as channels but also as signaling hubs that regulate cell behavior [7]. There are 21 known Cx isoforms in humans, classified by molecular weight and distribution in tissues [1]. Cxs differ mainly in the length of their CT that contains multiple phosphorylation sites; for example, human Cx43 has 33 phosphorylatable amino acid residues [8].

Cx43 is extensively studied due to its expression across the majority of tissues. It plays a key role in electrical communication in the heart and the brain [9]. Cx45 is also expressed in the heart and CNS neurons and is the first Cx to appear during embryonic development, marking early heart contractions [10]. In the heart, Cx45 localizes mainly in the conduction system, while Cx43 localizes in the working myocardium [11]. The role of Cx45 remains poorly explored, as knockout models are not viable postnatally.

Cx43 is described as one of the major players in tumorigenesis [12]. Tumor promoters often inhibit intercellular communication through GJs (GJIC), thereby promoting tumor growth, and the upregulation of Cx expression can suppress tumor formation [13]. However, contradictory data have also been reported, indicating that the inhibition of GJIC did not affect the proliferation and viability of Novikoff rat hepatoma cells expressing endogenous Cx43 and, under normal conditions, exhibiting large GJ conductance [14]. GJIC depends on several processes, including Cx synthesis, GJ channel formation, and pharmacological regulation.

Natural modulators such as resveratrol can induce a pro-apoptotic effect through the enhancement of GJIC [15,16]. Studies in rat liver epithelial cells showed that resveratrol can restore GJIC in a time- and dose-dependent manner [17]. Exposure time is a critical factor when evaluating the effects of resveratrol on cell viability. For example, treatment of liver stellate cells with 50 μM resveratrol for 24 h reduced cell viability due to oxidative stress; however, cytotoxicity decreased after 120 h, which suggests that surviving cells became more resistant to resveratrol-induced damage over time [18]. Alongside exposure time, resveratrol concentration is equally important. Studies in vitro use a wide concentration range of resveratrol, but the optimal dose in vivo requires further investigation in order to maximize its health benefits without toxicity. Resveratrol has been shown to produce anti-cancer effects in many in vivo and in vitro studies by inhibiting proliferation, inducing apoptosis, suppressing metastasis, and modulating the tumor microenvironment in colon, breast, prostate, and lung cancers [19]. It also enhances cancer cell sensitivity to chemotherapy and radiotherapy and can suppress multidrug resistance, making it a promising adjunct to conventional therapies [20,21]. Resveratrol is classified as a phytoestrogen due to its structural similarity to estrogen and its ability to interact with estrogen receptors (ER) [19]. It can bind to both ERα and ERβ as a mixed agonist/antagonist, influencing estrogen-responsive gene expression according to cell type, receptor subtype, and context [22]. For this reason, resveratrol should be used cautiously in cancer patients. However, the dominant biological activity of resveratrol is attributed to its potent antioxidant effects, through which resveratrol protects cells against oxidative stress-induced damage. Studies involving animal and cellular models demonstrate that resveratrol reduces reactive oxygen species production and lipid peroxidation while enhancing endogenous antioxidant enzyme activity across multiple disease models [23]. Resveratrol has been reported to prevent oxidative stress-induced cardiac hypertrophy by protecting liver kinase B1 and restoring activated protein kinase activity, suppressing pro-hypertrophic signaling in both in vitro cardiomyocytes and in vivo hypertensive rat models [24]. In a rat model of Parkinson’s disease induced by 6-hydroxydopamine, resveratrol demonstrated antioxidant effects by decreasing reactive oxygen species levels, reducing lipid peroxidation, and preserving glutathione content, alongside improved antioxidant enzyme activities in brain tissue [25].

Although resveratrol is most studied for its antioxidant properties, increasing evidence suggests that its effects on the cell extend beyond the regulation of redox balance alone. Oxidative status is closely linked to intercellular communication [26,27]; therefore, the influence of resveratrol on cell–cell communication, particularly through connexins and GJs, has become an important area of research. While much is known about the impact of resveratrol on Cx43 expression, data on its direct effect on Cx43 GJ conductance remain scarce. Resveratrol has been shown to enhance GJIC by upregulating Cx43 expression and function in various cell types. In retinal pigment epithelial cells under high glucose, resveratrol prevented Cx43 downregulation, restored GJIC, and reduced inflammation [28]. In melanoma, resveratrol increased Cx43 expression levels and GJIC, enhancing chemosensitivity [29]. Resveratrol also counteracted GJIC inhibition caused by tumor promoters or oxidative stress by blocking Cx43 and extracellular signal-regulated protein kinase 1/2 (ERK1/2) phosphorylation [30]. ERK1/2 phosphorylation is a crucial step in the mitogen-activated protein kinase (MAPK) pathway, playing a fundamental role in regulating key cellular processes such as proliferation, differentiation, survival, growth, metabolism, migration, and gene expression. The effects of resveratrol on Cx45-dependent GJIC are much less explored. While substantial evidence exists on the impact of resveratrol on ERK1/2 signaling, studies directly linking resveratrol, ERK1/2, and Cx45 are lacking in the current literature. In HeLa cells expressing exogenous Cx45, resveratrol, vitamin E, and tempol were found to reduce hemichannel opening. Although resveratrol appears to inhibit Cx45 hemichannel activity, likely due to its antioxidant properties through reducing free radical generation and potentially protecting against oxidative stress [31], there are currently no data on its effects on Cx45-mediated GJIC.

Previous studies have mostly focused on changes in Cx phosphorylation, expression, or GJIC assessed by molecular assays or dye transfer methods, rather than direct electrophysiological measurements. Therefore, the aim of this study was to improve the understanding of the dose- and time-dependent effects of resveratrol in cells expressing different types of Cxs by combining several approaches, such as dual whole-cell patch-clamp, molecular modeling, time-lapse imaging, Western blotting (WB), and fluorescence microscopy.

## 2. Materials and Methods

### 2.1. Cell Lines and Culture Conditions

Experiments were conducted on HeLa cells (ATCC CCL-2) stably transfected with either mouse or rat wild-type Cx45 and Cx43, or with tagged enhanced green fluorescent protein (EGFP). Additionally, Novikoff rat hepatoma cells expressing endogenous Cx43 were used. All cell lines were generously provided by the late Dr. F. Bukauskas (Albert Einstein College of Medicine, New York, NY, USA). Cells were cultured in Dulbecco’s Modified Eagle Medium (DMEM) containing 10% fetal bovine serum and a mixture of 100 U/mL penicillin and 100 μg/mL streptomycin (Sigma-Aldrich, Steinheim, Germany) and maintained at 37 °C in a humidified atmosphere with 5% CO_2_. *trans*-resveratrol (Cat no. R5010) and all other reagents were purchased from Sigma-Aldrich.

### 2.2. Molecular Docking

Molecular docking was carried out as described previously [32]. The human Cx43 structure (PDB ID 7F94) [33], featuring the I164V mutation incorporated using ChimeraX software (v. 1.6.1) [34], was adapted to represent the rat Cx43 (rCx43) structure for molecular docking. The resultant Cx43 model underwent validation by ProSA [35] and Procheck (v. 3.5.4) [36]. The mouse Cx45 (mCx45) structure was modeled with AlphaFold 2 and validated with an internal validation score [37]. The 3D molecular structure of resveratrol was downloaded from the PubChem database [38]. Molecular docking was performed using Utilizing Smina software (v. Oct 15 2019) [39] alongside a customized Vina scoring function [40]. To prevent the placement of symmetric docking conformations within neighboring subunits, the docking software was configured to use two adjacent Cx43 hemichannel subunits. The random seed was steadily set to one. The most favorable nine docking conformations were recorded. Two-dimensional docking images were created with LigPlot+ (v. 2.2.9) software [41].

### 2.3. Electrophysiological Measurements

For electrophysiological recordings, cells grown on glass coverslips were transferred to a continuously perfused experimental chamber mounted on the stage of an inverted microscope Olympus IX81 (Olympus Europa Holding GmbH, Hamburg, Germany) equipped with an Orca-R2 cooled digital camera (Hamamatsu Photonics K.K., Hamamatsu, Japan). Junctional conductance g_j_ between adjacent cells was determined using the dual whole-cell patch-clamp technique, as previously described [42]. Before measuring the initial g_j_, cells that were incubated with resveratrol for 24 h were perfused for 30 min with a control solution.

### 2.4. Flow Cytometry Assay

Initially, 5 × 10^4^ cells were seeded in a 12-well plate per well in 1 mL of complete medium. Cells were treated with resveratrol for 24 h, then washed twice with PBS, trypsinized, and collected by centrifugation. Subsequently, cells were maintained with Guava Nexin^®^ Reagent (Millipore, Burlington, MA, USA) at room temperature for 20 min according to the manufacturer’s protocol. Analysis of the samples was performed using a Guava^®^ easyCyte™ HT flow cytometer (Millipore, USA), and the data were analyzed by GuavaSoft 2.2.3 InCyte software.

### 2.5. Western Blotting

Cells were lysed on ice using cell extraction buffer (Invitrogen, Waltham, MA, USA) containing 1 mM PMSF (Abcam, Cambridge, UK), 20 μL/mL protease inhibitor cocktail, and 10 μL/mL phosphatase inhibitor cocktail (both from Sigma-Aldrich, Steinheim, Germany) for 30 min. Samples were centrifuged at 4 °C for 10 min at 13,000 rpm. Total protein levels were quantified with a Qubit^®^ protein assay kit and a Qubit 3.0 fluorometer (Invitrogen, USA). Protein samples were resolved by Bolt™ 4–12% Bis-Tris plus gels (Invitrogen, USA) and transferred onto PVDF membranes (Millipore, USA), then incubated for 1 h with primary antibodies against Connexin-45 (40–7000; Invitrogen, USA), Connexin-43 (C6219; Sigma-Aldrich, Steinheim, Germany), phospho-Connexin-43 (Ser368) (PA5-118544; Invitrogen, USA), phospho-Connexin-43 (Ser282) (PA5-64641; Invitrogen, USA), phospho-Connexin-43 (Ser279) (PA5-64640; Invitrogen, USA), phospho-Connexin-43 (Ser373) (PA5-64670; Invitrogen, USA), ERK1/ERK2 (MA5-15134; Invitrogen, USA), phospho-ERK1/ERK2 (MA5-15173; Invitrogen, USA), and GAPDH (AM4300; Invitrogen, USA). Proteins were detected with the WesternBreeze^®^ chemiluminescent kit (Invitrogen, USA) according to the manufacturer’s protocol. Band signals were captured using the G:Box Chemi Gel Documentation system (Syngene, Baltimore, MD, USA).

### 2.6. Immunocytochemistry

Cells were cultured on sterile glass coverslips and fixed for 15 min at room temperature in 4% paraformaldehyde. Cells were washed and then permeabilized with 0.2% Triton X-100 in PBS for 3 min. Next, samples were exposed to the primary antibodies for 1 h at 37 °C, diluted in 1% BSA/PBS. After washing with 1% BSA/PBS, cells were maintained at 37 °C for 30 min with species-specific secondary antibodies conjugated to distinct fluorophores: Alexa Fluor 488 and Alexa Fluor 647 (all at 1:125, Life Technologies, Carlsbad, CA, USA). F-actin was labeled by incubating cells with Alexa Fluor™ 594 phalloidin (A12381; Invitrogen, USA) for 30 min. Coverslips were mounted using a Vectashield Mounting Medium with DAPI (Vector Laboratories, Newark, CA, USA). The samples were analyzed with an Olympus IX83 inverted confocal microscope (Olympus Europa Holding GmbH, Hamburg, Germany). For the estimation of GJ plaque size, 3D images were obtained as Z-stacks at 0.25 μm intervals. The estimation of GJ plaque surface area (µm^2^) was performed using Fiji/ImageJ 3D Object Counter software (Version 1.54), where at least 3 zones were analyzed from 3 biological replicates, and no less than 100 GJ plaques were analyzed for each condition.

### 2.7. Live Cell and Time-Lapse Imaging

Live-cell imaging was carried out at 37 °C with 5% CO_2_ using an incubation system INUBG2E-ONICS (Tokai Hit, Shizuoka-ken, Japan) with an incubator, mounted on the stage of an Olympus IX83 inverted confocal microscope (Olympus Europa Holding GmbH, Hamburg, Germany). HeLa cells were seeded onto glass-bottom dishes and maintained in full DMEM. For the connexin/lysosome colocalization assay, HeLa Cx45-EGFP and HeLa Cx43-EGFP cells were labeled with SiR-Lyso 1 h prior to imaging. Data were assessed from 3 biological replicates. AGJ-lysosome quantification was performed by analyzing 20 AGJs per replicate. Cx45/lysosome colocalization was assessed by analyzing 20 cells per replicate. Analysis was performed using Fiji/ImageJ Colocalization software (Version 1.54). For cytoskeletal images, HeLa WT, HeLa Cx43, Hela Cx45, and HeLa Cx43 EGFP cells were labeled with SiR-Actin and 4-580CP-LTX fluorescent probes. Time-lapse imaging for GJ plaque internalization was automatically set to be taken at 10 min intervals. Images were collected as Z-stacks using 0.5–1 μm steps. Data were assessed from 4 biological replicates.

### 2.8. Statistical Analysis

Experimental data are presented as means ± standard error of the mean. Statistical comparisons were conducted using a two-tailed Student’s *t*-test, with *p*-values  <  0.05 considered statistically significant. All statistical analyses were performed with SigmaPlot version 12.0 software.

## 3. Results

### 3.1. Resveratrol Differently Modulates Cx43 and Cx45 GJ Conductance

**Figure 1:**
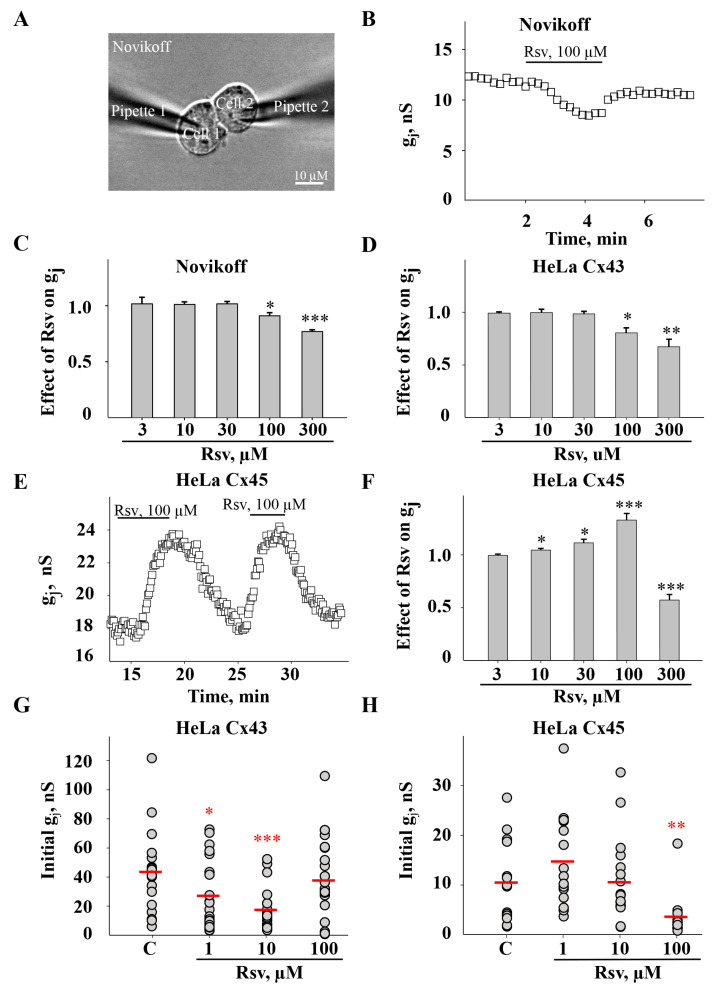
The effect of resveratrol on Cx43 and Cx45 GJ conductance. (**A**) Junctional conductance (g_j_) between contiguous cells was measured using the dual whole-cell patch-clamp technique. Effect of resveratrol on Cx43 g_j_ in Novikoff cells (**B**,**C**) and HeLa Cx43 cells (**D**). Effect of resveratrol on g_j_ between HeLa Cx45 cells (**E**,**F**) (n = 4). Initial Cx43 (**G**) and Cx45 (**H**) GJ conductance after preincubation of HeLa cells with resveratrol for 24 h. Data were assessed from at least 3 biological replicates. * *p* < 0.05; ** *p* < 0.01; *** *p* < 0.001 vs. Control. Control—C; resveratrol—Rsv. Red horizontal bars represent the mean values.

We used the dual whole-cell patch-clamp technique (Figure 1A) to assess the effect of resveratrol on GJ conductance (g_j_) between Novikoff cells expressing endogenous Cx43 and HeLa cells expressing exogenous Cx43 and Cx45. Resveratrol had no effect on Cx43 GJ g_j_ at low doses (3, 10, 30 µM) and inhibited it at higher concentrations (100 and 300 µM) in both Novikoff and HeLa Cx43 cells (Figure 1B–D). In contrast, in HeLa cells expressing Cx45, resveratrol stimulated the g_j_ at 10 and 100 µM but inhibited it at 300 µM (Figure 1E,F). To assess the long-term effect of resveratrol, we preincubated cells with it for 24 h and measured the initial Cx43 and Cx45 GJ g_j_ in HeLa cell pairs right after the initiation of patch-clamp conditions. Surprisingly, a decrease in initial Cx43 GJ g_j_ was observed after treatment with resveratrol at lower concentrations (1 and 10 µM), while 100 µM had no effect (Figure 1G). Meanwhile, resveratrol reduced initial Cx45 GJ g_j_ only after incubation with 100 µM (Figure 1H). The effect of 300 µM resveratrol on g_j_ after cell incubation for 24 h could not be precisely assessed, as it drastically affected cell viability.

### 3.2. Docking of Resveratrol to Cx43 and Cx45

**Figure 2:**
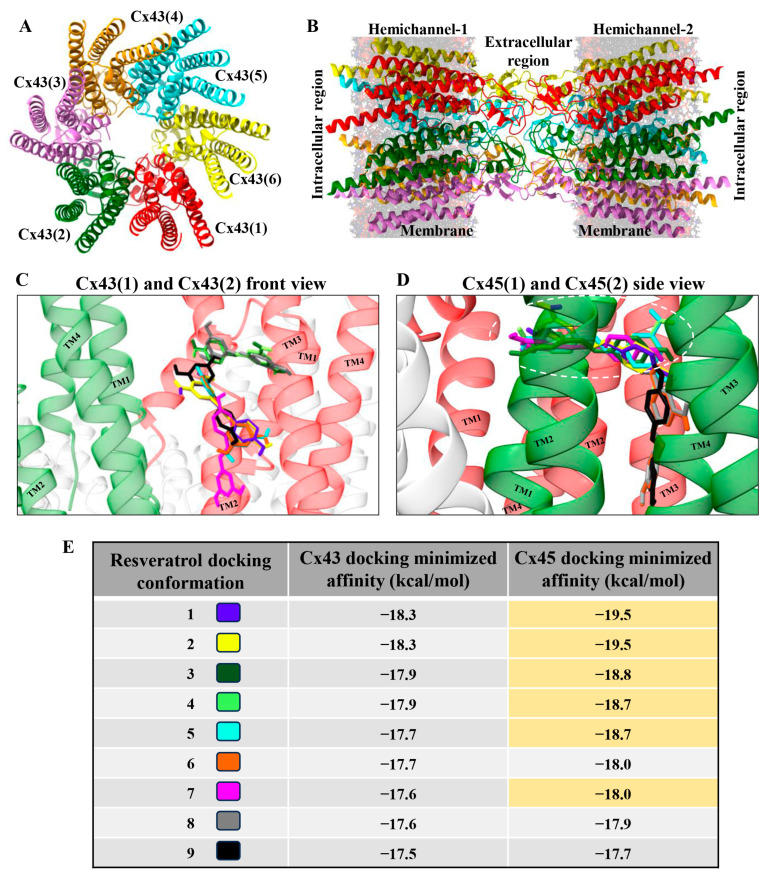
Interaction of resveratrol with Cx43 and Cx45 subunits. Top (**A**) and front (**B**) view of Cx43 gap junction presented without intracellular N- and C-termini of connexins (Cx43 subunit number is indicated in parentheses). The nine best docking conformations of resveratrol on Cx43 (**C**) and Cx45 (**D**) subunits (TM1-TM4 are transmembrane domains of connexins). Minimized docking affinities of resveratrol to each of nine docking sites on Cx43 and Cx45 (**E**). A more negative value indicates a stronger interaction between resveratrol and connexin. Docking sites situated in the niche between Cx45 subunits (encircled by a white dashed line in (**D**)) exhibit higher affinities to resveratrol (highlighted in yellow in (**E**)).

As shown in Figure 1, resveratrol exerted different effects on Cx43 and Cx45 GJ g_j_. At low concentrations, it stimulated only Cx45 GJ g_j_ but at higher doses it inhibited both Cx45 and Cx43 GJs. Therefore, we presumed that Cx45 should exhibit two different docking profiles, the first including high-affinity docking sites responsible for the stimulation of g_j_, and the second including low-affinity docking sites responsible for the inhibition of g_j_. To verify this hypothesis, we performed molecular docking experiments. Figure 2A represents a top view of the GJ hemichannel composed of six Cx43 subunits (subunit number is indicated in parentheses), and Figure 2B shows a side view of the GJ channel composed of two apposed hemichannels. We analyzed the nine most favorable docking conformations and found that they all localized on the outer surface of Cx43 TM2 and TM3 (Figure 2C and Supplementary Movie S1) with minimized docking affinities varying from −18.3 to −17.5 kcal/mol (more negative values indicate stronger interaction between resveratrol and connexin) (Figure 2E). However, only three docking sites on Cx45 localized on its outer surface with minimized affinities from −18.0 to −17.7 kcal/mol, while the remaining six ones were situated in the niche between two neighboring Cx45 subunits towards the channel pore (Figure 2D and Supplementary Movie S2), and the docking affinities of five of them varied from −19.5 to −18.7 kcal/mol (highlighted in yellow in Figure 2E). This finding suggests that higher-affinity docking locations in the niche that are absent in Cx43 could be responsible for Cx45 GJ g_j_ stimulation, with lower concentrations of resveratrol observed in dual whole-cell patch-clamp experiments (see Figure 1), while lower-affinity docking sites that are similar in both Cxs are responsible for g_j_ inhibition. The most favorable amino acid residues for docking resveratrol in the niche were Ile82 and Thr86 in Cx45(1) TM2 and Val27 in Cx45(2) TM1 (highlighted in Supplementary Table S1; also see Supplementary Movie S2).

### 3.3. The Effect of Resveratrol on Cx43 and Cx45 Expression and GJ Plaque Size

**Figure 3:**
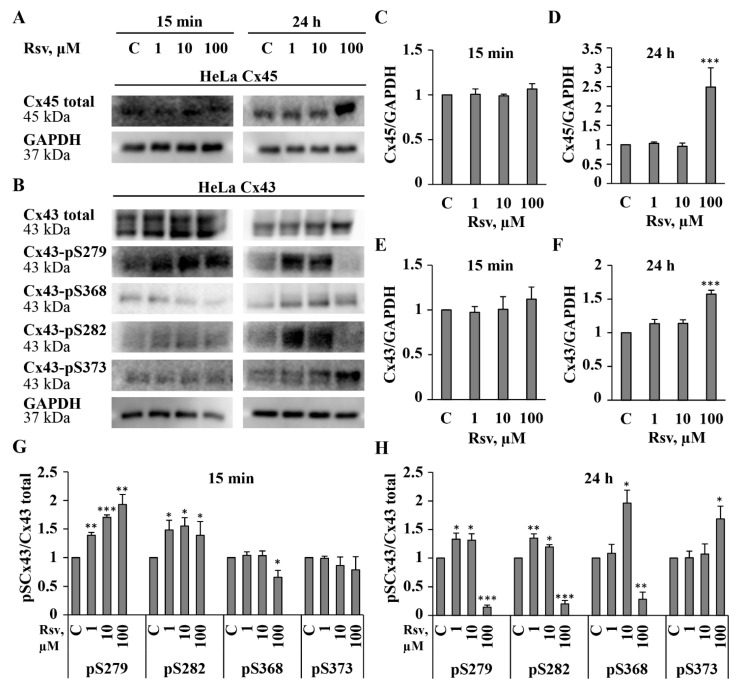
Effect of resveratrol on Cx45 and Cx43 protein expression. Representative WBs showing the total levels of Cx45 (**A**) and Cx43 together with the ratio of total Cx43 to phosphorylated Cx43 (**B**), following resveratrol treatment at indicated concentrations for 15 min and 24 h. Summary of the effect of resveratrol on the total expression of Cx45 (**C**,**D**) and Cx43 (**E**,**F**) and Cx43 phosphorylated forms (**G**,**H**). WBs from each of the 3 replicates are presented in Supplementary Figures S1–S3. * *p* < 0.05; ** *p* < 0.01; *** *p* < 0.001 vs. Control.

The obtained results have demonstrated that resveratrol induced fast, reversible, Cx type-dependent effects on g_j_ (Figure 1A–F) and an inhibition of initial g_j_ after preincubation for 24 h (Figure 1G,H). Therefore, we further examined the effect of preincubation with resveratrol on Cx expression and GJ plaque size. Quantitative evaluation of Cx43 and Cx45 proteins was performed after 15 min (time needed to reach the steady-state effect in patch-clamp measurements) and after 24 h of resveratrol treatment. After 15 min from the application of resveratrol at any concentration, total Cx45 and Cx43 protein levels remained unchanged. After 24 h, 1 and 10 μM of resveratrol also had no effect; however, 100 μM increased Cx45 and Cx43 levels by approximately 2.5- and 1.6-fold, respectively (Figure 3A–F). It is well established that the synthesis, assembly, and function of GJs are regulated by phosphorylation of Cxs at specific sites [8]. Therefore, we assessed changes in the phosphorylation of Cx43 at serine residues S279, S282, S368, and S373, which have been shown to be involved in the regulation of GJ expression and function [2]. The obtained results were well aligned with our patch-clamp and immunocytochemical findings. Phosphorylation at S282 and S279 is known to inhibit Cx43 channel conductance, whereas phosphorylation at S373 is associated with increased GJ plaque size [43]. Consistent with this, a decrease in Cx43 GJ g_j_ was accompanied by elevated levels of pS282 and pS279 at both time points. Additionally, an increase in pS373 phosphorylation correlated with the formation of larger Cx43 plaques after 24 h of treatment with 100 μM of resveratrol (see data below). Another well-known phosphorylation site involved in GJIC regulation is S368 [44]. Its phosphorylation affects channel permeability and reduces GJIC between cardiomyocytes due to GJ internalization [45]. We found that pS368 levels decreased after both 15 min and 24 h treatment with 100 μM resveratrol, while a 2-fold increase in pS368 was observed after 24 h of treatment with 10 μM resveratrol (Figure 3B,G,H). In contrast to Cx43, only a few phosphorylation sites of Cx45 are documented [2], and no phosphorylation-dependent changes in Cx45 GJ g_j_ have been demonstrated, so far. Overall, these data show that the complexity of the responses depends on the nature, strength, and duration of the stimulus. In addition, GJ gating and/or Cx trafficking may be regulated through second messenger pathways and the combined actions of multiple kinases and sequential phosphorylation events of Cx43 CT [43].

**Figure 4:**
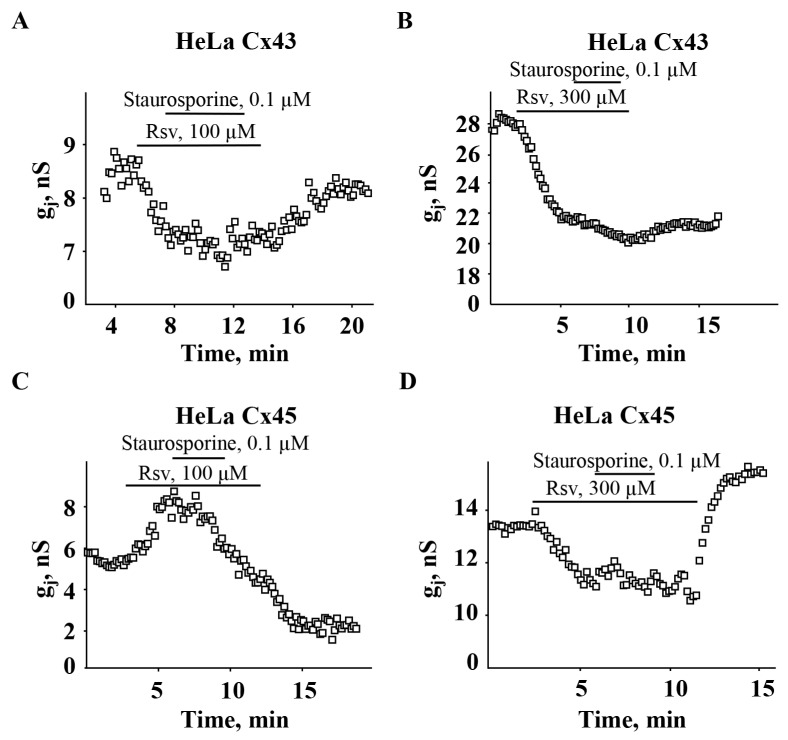
The effect of resveratrol on Cx43 and Cx45 GJ conductance does not depend on kinase activity. The effect of resveratrol on Cx43 (**A**,**B**) and Cx45 (**C**,**D**) GJ g_j_ is not abolished by staurosporine (n = 4 for each set of experiments).

The relationship between Cx45 phosphorylation and Cx45 GJ properties is basically underexplored due to the absence of specific antibodies. In the current study, we examined the effect of resveratrol on only the total expression of Cx45 (Figure 3A,C,D); however, in general, the impact of protein kinase activity on GJ conductance can be examined using kinase inhibitors. We have previously shown that the potency of certain compounds can be allosterically modulated by phosphorylation of Cx43 CT by Ca^2+^-regulated kinases [8]. The observation of resveratrol-induced changes in phosphorylation of Cx43 CT sites (Figure 3B,G,H) prompted us to verify whether the potency of resveratrol is phosphorylation-dependent. However, staurosporine, a nonselective kinase inhibitor, neither altered the inhibitory effect of resveratrol on Cx43 GJ g_j_ (Figure 4A,B) nor its stimulatory and inhibitory effect on Cx45 GJ g_j_ (Figure 4C,D).

**Figure 5:**
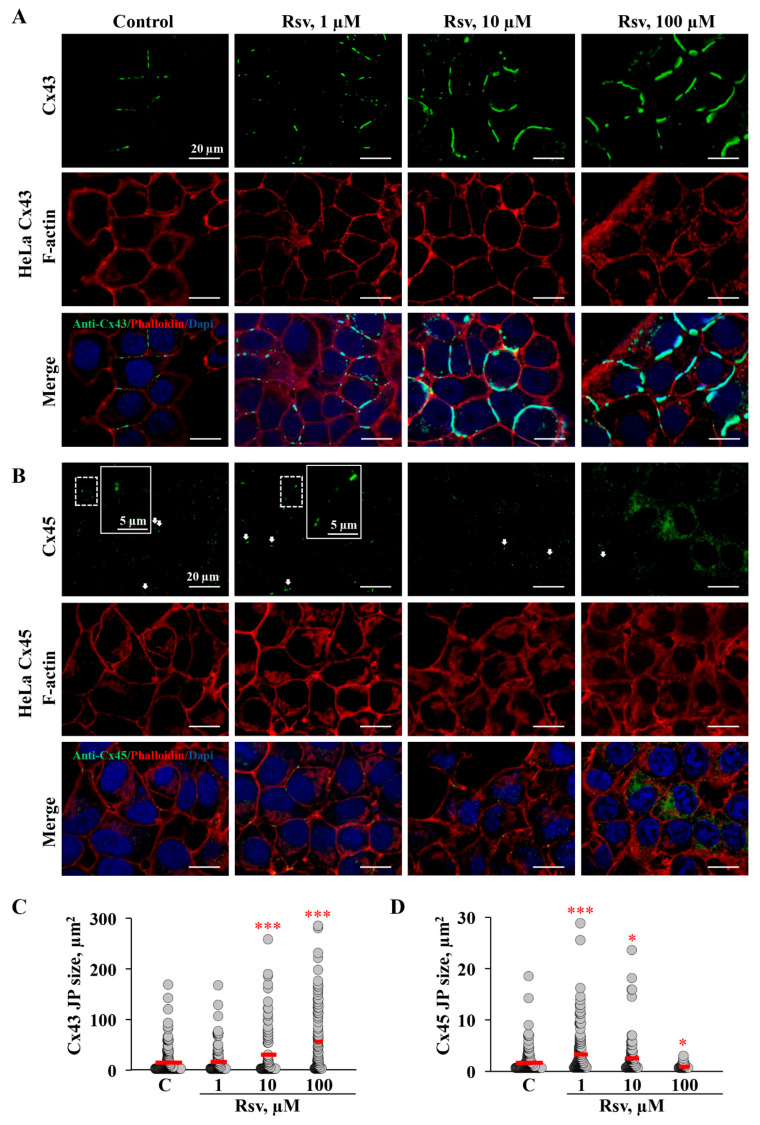
The effect of resveratrol on Cx43 and Cx45 GJ plaque size in HeLa cells. Typical immunofluorescence images of GJ plaques in HeLa Cx43 (**A**) and HeLa Cx45 (**B**) cells under control conditions and after 24 h treatment with resveratrol at the indicated concentrations. Scale bars are identical for all sub-figures. White arrows indicate GJ plaques. Summary of resveratrol’s effects on Cx43 (**C**) and Cx45 (**D**) GJ plaque size. * *p* < 0.05; *** *p* < 0.001 vs. Control. Red horizontal bars represent the mean values.

Further, we examined whether the application of resveratrol could induce changes in GJ plaque size. Although changes in Cx expression were observed only after 24 h treatment with 100 μM resveratrol in both HeLa Cx43 and HeLa Cx45 cells (Figure 3A–F), initial g_j_ was altered after application of 1 and 10 μM in HeLa Cx43 (Figure 1G). Exposure of HeLa Cx43 cells to 10 and 100 μM resveratrol for 24 h resulted in the formation of exceptionally large GJ plaques (Figure 5A,C). The estimated Cx43 GJ plaque size was 14.7 ± 1.8 µm^2^ under control conditions and increased to 30.4 ± 5.1 µm^2^ and 56.0 ± 6.5 µm^2^ following treatment with 10 and 100 μM resveratrol, respectively. Surprisingly, initial Cx43 GJ g_j_ was reduced after treatment with 1 and 10 µM resveratrol for 24 h (Figure 1G), while 10 μM increased the GJ plaque size, and 1 μM had no effect on it. We rather expected a reduction in GJ plaque size; however, these results could be in part explained by increased phosphorylation at S279 and S282 (Figure 3), which might be responsible for the reduced Cx43 GJ g_j_ (Figure 1G).

Cx45 GJ plaque size increased after 1 and 10 μM but decreased after treatment with 100 μM resveratrol. Cx45 formed much smaller GJ plaques compared to Cx43. The sizes of Cx45 GJ plaques in control and after treatment with 1, 10, and 100 µM resveratrol were 1.7 ± 0.2, 3.3 ± 0.3, 2.5 ± 0.3, and 0.9 ± 0.1 µm^2^, respectively (Figure 5D). Immunofluorescence analysis revealed that after 24 h treatment with 100 μM resveratrol, Cx45 not only formed smaller GJ plaques, but also was more abundant in the cytoplasm (Figure 5B,D). These results could explain a decrease in electrical coupling, as shown above (Figure 1H).

### 3.4. Resveratrol Modulates ERK1/2 Activation and Cellular Viability

**Figure 6:**
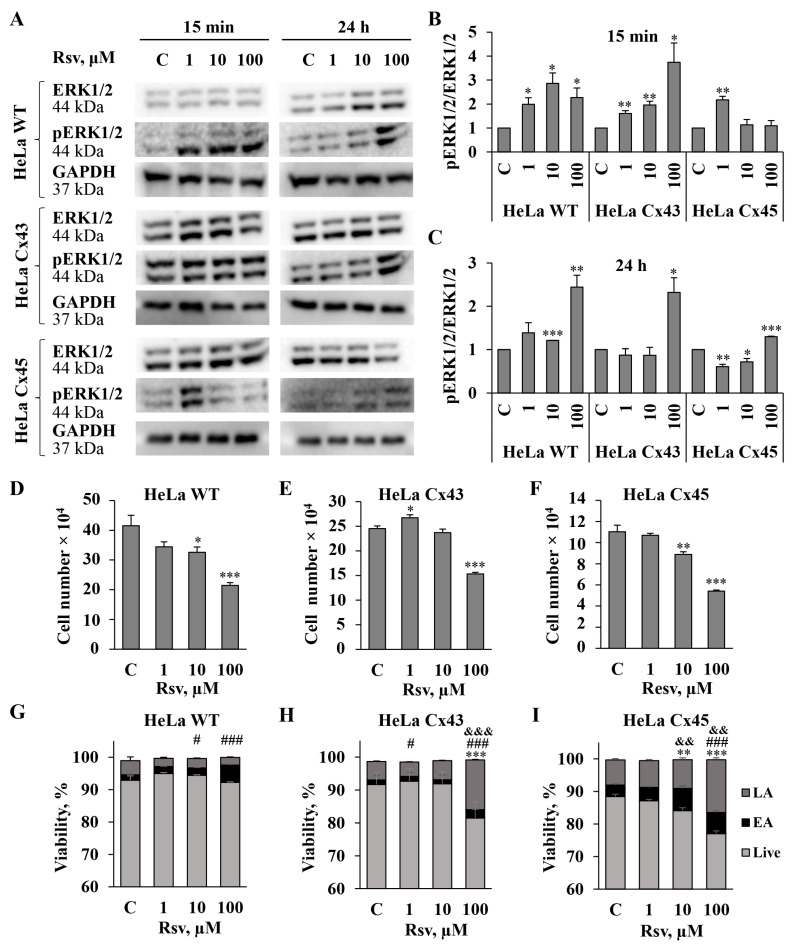
The effect of resveratrol on ERK1/2 and phosphorylated ERK1/2 levels and HeLa WT, HeLa Cx43, and HeLa Cx45 cell proliferation and viability. (**A**) Representative WBs showing expression of ERK1/2 along with its phosphorylated form after resveratrol treatment for 15 min and 24 h. (**B**,**C**) Summary of the effect of resveratrol on the expression of ERK1/2 and pERK1/2. WBs from each of the 3 replicates are presented in Supplementary Figures S1–S4. Resveratrol-induced changes in proliferation of HeLa WT (**D**), HeLa Cx43 (**E**), and HeLa Cx45 (**F**) cells after 24 h of treatment at the indicated concentrations. The effect of resveratrol on the viability of HeLa WT (**G**), HeLa Cx43 (**H**), and HeLa Cx45 (**I**) cells compared to control (n = 3). *^,#^ *p* < 0.05; **^,&&^ *p* < 0.01; ***^,###,&&&^ *p* < 0.001. *—for live cells (Live), ^#^—early apoptosis (EA), ^&^—late apoptosis (LA).

Phosphorylation of ERK1/2 is a key event in the MAPK signaling pathway, influencing cell survival, proliferation, and death [46,47]. The effect of resveratrol may be either activatory or inhibitory, depending on the disease or cell type [48,49]. It has been shown that the enhancement of Cx43 expression and GJIC by resveratrol involves MAPK and ERK1/2 signaling pathways [29,30]. It is known that resveratrol inhibits ERK1/2 phosphorylation and reduces HeLa cell adhesion [50]. In some other cancer cells, such as mouse melanoma and human renal cell carcinoma, resveratrol targets the ERK1/2 pathway and exerts anti-proliferative, anti-migratory, or pro-apoptotic effects via the modulation of upstream regulators [51,52]. Research on the interplay between GJIC and ERK has mostly focused on Cx43, highlighting that ERK-mediated phosphorylation modulates the gating, assembly, and turnover of Cx43 GJ channels. Phosphorylation of Cx43 CT on S279 and S282 has been reported as ERK1/2-dependent [2,43,53]. As demonstrated in Figure 3, resveratrol affects the phosphorylation of several Cx43 residues, including S279 and S282. Due to the aforementioned observations, we examined the effect of resveratrol on pERK1/2/ERK1/2 and the viability of communication-deficient HeLa WT cells and HeLa cells expressing exogenous Cx43 and Cx45 (Figure 6). We examined changes in protein expression after 15 min and 24 h treatment with resveratrol, but the results of the viability assay are shown only for the 24 h time point, because 15 min exposure did not cause any changes in viability. In HeLa WT and HeLa Cx43 cells, all concentrations of resveratrol increased pERK1/2 levels after 15 min treatment, whereas in HeLa Cx45 cells, only 1 µM concentration increased pERK1/2 levels. Treatment with 1 µM resveratrol for 24 h affected only HeLa Cx45 cells by decreasing pERK1/2 levels. Resveratrol at a 10 µM concentration increased pERK1/2 expression only in HeLa WT cells, decreased it in HeLa Cx45 cells, and had no effect in HeLa Cx43 cells. Treatment with 100 µM resveratrol for 24 h upregulated pERK1/2 activity in all HeLa cell lines, concomitantly suppressing cell proliferation and viability. Resveratrol (100 µM) inhibited proliferation by 48%, 38%, and 51% in HeLa WT, HeLa Cx43, and HeLa Cx45 cells, respectively (Figure 6D–F). HeLa Cx43 and Cx45 cells were more sensitive to 100 µM resveratrol treatment since their viability dropped to 81% and 76%, respectively, while the viability of HeLa WT cells remained near the control level (Figure 6G–I). After treatment with resveratrol, the majority of non-viable HeLa WT, HeLa Cx43, and HeLa Cx45 cells were found in a late apoptotic state.

Taken together, our results show that pERK1/2 activation by resveratrol treatment correlates with a decrease in cell viability and proliferation.

### 3.5. Impact of Resveratrol on Cytoskeletal Structure and Gap Junction Degradation

**Figure 7:**
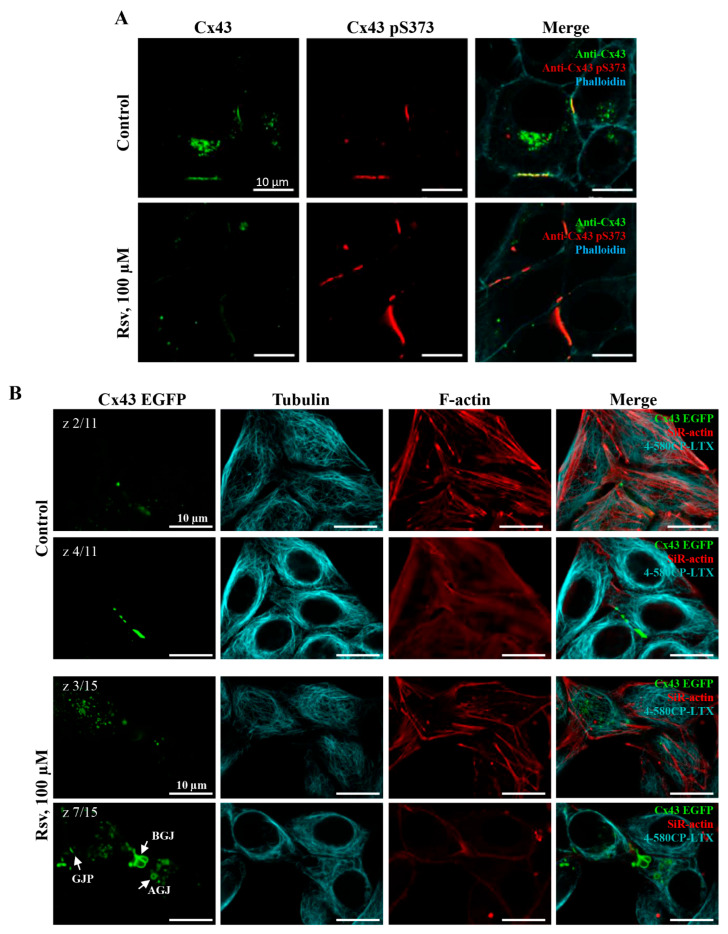
The effect of 24 h resveratrol treatment on F-actin and α-tubulin network in HeLa Cx43 and HeLa Cx43-EGFP cells. (**A**) Immunofluorescence images of Cx43 and Cx43-pS373. After resveratrol treatment, the enlarged Cx43 GJ plaques exhibit increased levels of pS373. (**B**) Hela Cx43-EGFP live cells labeled with F-actin (SiR-actin) and α-tubulin (4-580CP-LTX) probes and imaged at different z-planes. Resveratrol does not visibly affect the GJ internalization since GJs at different internalization stages (intact GJ plaques (GJP), budded and starting to internalize GJs (BGJ), and fully internalized annular gap junctions (AGJ)) are present. Scale bars are identical for all sub-figures.

Our results indicate that GJ plaque size increased following 24 h treatment with resveratrol at 10 or even at 1 µM concentration in HeLa Cx45 cells (Figure 5), while elevation in Cx43 and Cx45 expression was observed only after the application of 100 µM resveratrol (Figure 3A–F). Interestingly, the elevation of Cx43-pS373, which coincides with Cx43 GJ plaque enlargement [43], was observed by WB and immunofluorescence assays only after 24 h treatment with 100 µM (Figure 3 and Figure 7A). This suggests that other Cx43 phosphorylation sites and/or intermediate pathways could be responsible for this effect.

In the following step, we set out to examine whether resveratrol’s effects on GJ turnover are related to its effect on cytoskeletal integrity. We performed imaging of HeLa Cx43, HeLa Cx45, and HeLa WT live cells labeled with F-actin and α-tubulin fluorescent probes (Supplementary Figure S5). Also, HeLa cells expressing Cx43 tagged with EGFP (Cx43-EGFP) were used for time-lapse imaging of GJ plaque dynamics (Figure 7B). Resveratrol treatment did not cause an obvious disarrangement of actin and microtubule cytoskeleton network, and consequently, regular internalization of GJ plaques and formation of annular junctions were supposed to persist.

**Figure 8:**
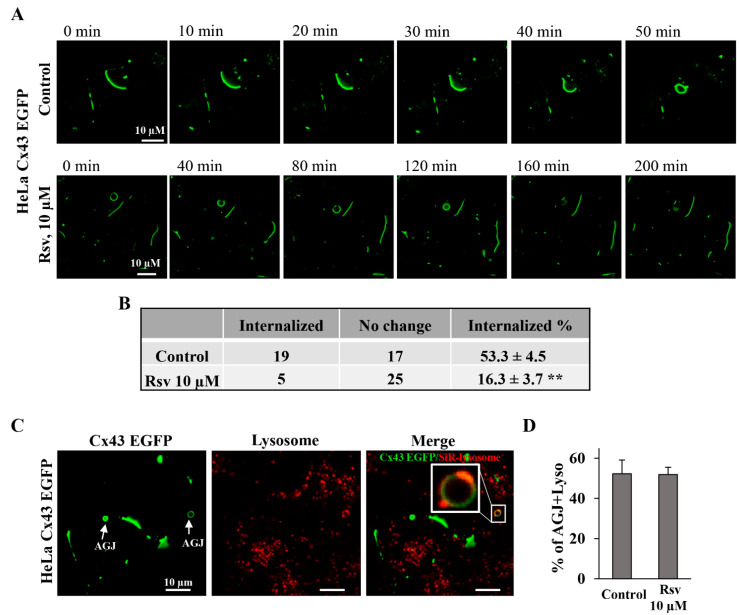

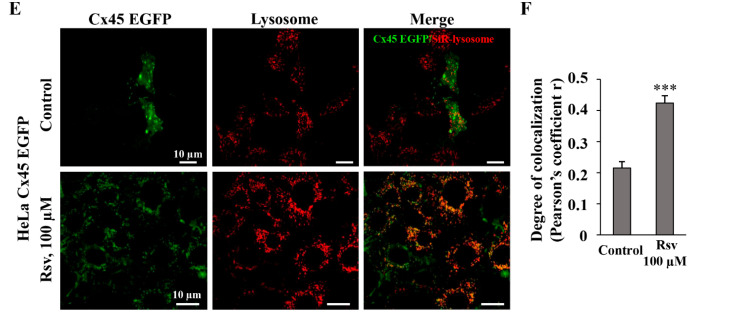
The effect of resveratrol on Cx43 and Cx45 degradation. (**A**) Time-lapse images of HeLa Cx43-EGFP in control conditions and after treatment with resveratrol (10 µM, 24 h). (**B**) Summary of Cx43 GJ plaque internalization rate. (**C**) Typical image of AGJ co-localization with lysosomes. (**D**) Summary of AGJ co-localization with lysosomes in control and resveratrol treated Hela Cx43-EGFP cells. (**E**) Typical images of Cx45 co-localization with lysosomes. (**F**) Summary of Cx45 co-localization with lysosomes in control and resveratrol treated Hela Cx45-EGFP cells. Data represented from at least 3 independent experiments. ** *p* < 0.01; *** *p* < 0.001 vs. Control. Scale bars are identical for all sub-figures.

Next, we carried out time-lapse imaging to monitor the internalization of Cx43 GJ plaques. Some studies suggest that stress may slow down the internalization of Cx43 plaques [54]. It has been reported that the presence of GJ at the plasma membrane persists for up to 5 h [55]. Since it is virtually impossible to monitor the full life-time of GJ plaque starting from the exact zero-time of its formation until complete AGJ formation, we relied on statistics and performed time-lapse imaging of randomly selected cell pairs with GJ plaques and measured the number of internalized GJ plaques during 210 min recording under control conditions and after treatment with 10 µM resveratrol for 24 h (Figure 8A). The duration of the time-lapse experiments was selected because, under control conditions, Cx43 GJ plaques typically disassembled within a 191 ± 20 min (n = 30) period. Our findings indicate that resveratrol stabilized Cx43 plaques, leading to a 3-fold decrease in the internalization rate (Figure 8B). For these experiments, we chose a lower resveratrol concentration (10 µM) since it did not affect cell viability and proliferation (Figure 6D–I).

Furthermore, we examined the possible mechanism underlying retarded Cx43 degradation. Cx43 is regularly internalized from the plasma membrane and directed toward degradation, mainly through the endosome–lysosomal pathway. During this process, GJs are internalized, and AGJs are formed and transported for endosomal degradation [56]. We estimated the number of AGJs fused with lysosomes (Figure 8C) in control and in resveratrol-treated HeLa Cx43-EGFP cells and did not detect any changes in Cx43 and lysosome co-localization (Figure 8D).

Resveratrol at concentrations of 10 and 100 µM increased the Cx43 GJ plaque size after 24 h (Figure 5A,C) with a significant increase in total Cx43 expression only at a concentration of 100 µM (Figure 3B,D). In contrast, the Cx45 GJ plaque size was already significantly increased after treatment with 1 µM resveratrol; however, in contrast to Cx43, 100 µM resveratrol strongly reduced the Cx45 GJ size, although total Cx45 expression increased by 2.5-fold, like in the case of Cx43. Importantly, a decrease in the Cx45 GJ plaque size and g_j_ (Figure 1H) was followed by increased Cx45 degradation, as assessed by Cx45 and lysosome co-localization, which increased by ~2-fold (Figure 8E,F). Pearson’s correlation coefficient was used to quantify the degree of Cx45 and lysosome co-localization due to the fact that Cx45 forms very small GJ plaques (Figure 5) and inestimable AGJs compared to Cx43. However, the reasons why total Cx45 expression increases despite enhanced Cx45 degradation remain incompletely understood. We can only speculate that under stress conditions, when Cx45 GJ plaque internalization and degradation are accelerated, some compensatory mechanisms are activated to stimulate Cx45 synthesis, but not new GJ plaque formation, which is a much more complex process involving connexin oligomerization, hemichannel trafficking to the membrane, and docking of apposed hemichannels.

## 4. Discussion

In the present study, we found that resveratrol induced time- and dose-dependent changes in Cx43 and Cx45 expression, phosphorylation, GJ plaque size and conduction, and HeLa cell viability. Comparative analysis of Cx43 and Cx45 revealed that resveratrol suppressed GJIC in Cx43-expressing cells but enhanced it in Cx45-expressing cells (Figure 1). The dual effect could be at least in part explained by molecular docking experiments, which showed that Cx45 exhibited two different docking profiles of higher and lower affinity, while only the latter was observed in Cx43 (Figure 2). GJIC modulation is often linked with changes in connexin expression levels and GJ plaque size [57]. However, usually only a small part of the channels composing GJ plaques are functional, as has been shown for Cx43 [58], Cx45 [59], Cx57 [60], and Cx36 [61]. Therefore, the increase in GJ plaque size may not guarantee an increase in its total conductance. Long-term (24 h) treatment with resveratrol caused a reduction in initial g_j_ (Figure 1), but unexpectedly, this effect was followed by an increase in GJ plaque size in HeLa Cx43 cells (Figure 5), suggesting that the functional efficiency of GJ plaques was decreased. In HeLa Cx45 cells, the initial g_j_ remained unchanged (Figure 1), while GJ plaque size showed a tendency to increase (Figure 5). Changes in GJ channel conductance, along with the modulation of connexin expression, GJ plaque size, and turnover, are a complex process with phosphorylation being one of the most important regulators. For example, phosphorylation at S279/282 is linked to reduced GJIC, pS373 increases the amount of Cx43 at the plasma membrane and enhances the size of GJ plaques, while phosphorylation at S368 is involved in the dynamic turnover of GJ, influencing their assembly, internalization, and degradation [43]. Our findings mostly correspond to the previously published data, as the reduction in Cx43 GJ conductance (Figure 1) was accompanied by increased levels of Cx43 pS282 and pS279 (Figure 3), while phosphorylation at S373 (Figure 3) likely contributed to the formation of larger Cx43 plaques (Figure 5 and Figure 7).

GJIC efficiency may depend on Cx43 phosphorylation that affects the trafficking of hemichannels, their docking to form GJ channels, GJ aggregation into GJ plaques, and plaque turnover [43,62]. Most connexins are phosphorylated at specific sites by multiple kinases, and interplay between kinases may determine the ultimate cellular responses [8,63]. It was demonstrated that resveratrol modulates Cx43 phosphorylation via MAPK and phosphoinositide 3 kinase (PI3K)/protein kinase B (Akt) activation [64,65,66]. ERK1/2 phosphorylation is a central step in the MAPK pathway, regulating cell survival, proliferation, and death. Resveratrol modulates this pathway in a context-dependent manner, either activating or inhibiting it depending on the disease or cell type [52,67]. We found that ERK1/2 activation is associated with cell viability, as after treatment with the highest used resveratrol concentration (24 h, 100 µM), pERK1/2 levels peaked, while viability decreased (Figure 6). Resveratrol caused greater reduction in the viability of Cx43- and Cx45-expressing HeLa cells compared with HeLa WT cells (Figure 6). These results resemble the findings of other studies, where experiments with HeLa cells lacking exogenous Cxs and expressing Cx37, Cx40, and Cx43 showed that the pro-apoptotic effect of GJIC varied based on a connexin type, and HeLa Cx43 cells were most sensitive to streptonigrin-induced apoptosis. Moreover, the authors suggested that apoptosis was promoted by Cx43 and Cx40, but not by Cx37, via GJ transfer of IP_3_-dependent pro-apoptotic signals [68]. Our important finding was that treatment with 1 µM resveratrol increased the proliferation of HeLa Cx43 cells (Figure 6). This observation suggests that resveratrol may exert a concentration-dependent biphasic effect, i.e., lower concentrations could promote cell proliferation, while higher concentrations might inhibit it. Many polyphenolic extracts and pure polyphenols, such as quercetin and curcumin, tend to induce apoptosis and reduce cancer cell viability, in a dose-dependent manner, while having little or no cytotoxic effect on normal cells [69,70,71]. Resveratrol displays a dual action as well, being cytoprotective in healthy cells and cytotoxic in cancer cells [72,73]. Interestingly, a proliferation-promoting effect was demonstrated in T47D human breast cancer cells, explained by phytoestrogenic properties of resveratrol [74]. Usually, resveratrol acts as a tumor suppressor, but in certain estrogen-sensitive human breast cancer cells depending on ER isoforms or response elements, it can function as an estrogen agonist, thus promoting cell growth.

In addition to the above described mechanisms, resveratrol has been shown to disrupt the tubulin network by inhibiting tubulin polymerization [75,76,77]. Also, resveratrol has been shown to affect the actin cytoskeleton in a concentration-dependent manner, disrupting it in MDA-MB-231 breast cancer cells [78], or promoting actin accumulation at injury sites in mouse C2C12 myoblasts [79]. The microtubule and actin cytoskeleton is important for hemichannel transport to the plasma membrane, for maintaining their proper localization, and for stabilizing GJ plaques [80]. Our data show that resveratrol did not disrupt the actin cytoskeleton or the tubulin network in HeLa cells (Figure 7), independent of the presence or type of connexin. Although we cannot exclude the possibility that other factors involved in Cx trafficking, such as the scaffold protein ZO-1 [81], motor protein like dynein [82], GTPase dynamin 2 [83], E-cadherin, or N-cadherin [84], could be affected by resveratrol.

The time-lapse imaging showed that resveratrol stabilized Cx43 plaques, leading to a three-fold decrease in the internalization rate compared to the control. This finding is in agreement with previous work showing that genotoxic stress reduces Cx43 plaque internalization in primary bovine corneal endothelium cells that have been transiently transfected with Cx43-GFP [54]. Plaque size and lifespan are closely associated with the lysosomal degradation pathway, and its disruption may affect GJ plaque stability [56]. We hypothesized that resveratrol could trigger lysosomal degradation; however, no obvious changes were observed. Moreover, we expected that an increase in GJ plaque size would result in enhanced GJIC; however, Cx43 GJ g_j_ was decreased after 24 h treatment with 10 µM resveratrol (Figure 1). Presumably, this phenomenon could be explained by our earlier observations that a large total number of channels in the GJ plaques is unrelated to the functional efficiency of the plaques, because only a small number of channels are functional, and the overload of GJ plaques with channels may even hamper their functionality.

Resveratrol was selected for this study due to its broad range of physiological effects, including cardioprotective, anti-inflammatory, and metabolic actions mediated through multiple signaling pathways. Although resveratrol exhibits significant biological activity in vitro at relatively high concentrations (≥100 µM), several studies, including our own, have reported biological effects at lower concentrations [85,86,87]. Notably, in our study, the effects of resveratrol were observed even at concentrations of 1–10 µM. These concentrations, however, are still considerably higher than the plasma levels typically achieved after oral intake of resveratrol supplements or resveratrol-containing products such as red wine, which typically remain below 0.02 µM [88,89]. These pharmacokinetic limitations reflect the rapid metabolism (a half-life of approximately 9 h) and clearance of resveratrol, resulting in a short-lived presence of biologically active concentrations in the circulation. According to previous reports, extensive metabolism of resveratrol in the intestine and hepatic metabolism leads to oral bioavailability of the parent compound of only about 1% [90]. However, due to its lipophilic nature, concentrations of resveratrol in tissues may exceed those observed in plasma [91]. This phenomenon has also been demonstrated in patients with colorectal cancer, supporting the colorectum as a potential target for chemoprevention by oral resveratrol intake [92]. Interestingly, some studies showed that the bioavailability of resveratrol can be enhanced by using more potent resveratrol analogs, such as SRT501 [93], or by employing advanced delivery systems, including liposomal encapsulation [94], nanoparticles, and nanoemulsions. These approaches improve solubility, stability, and systemic exposure, thereby increasing the therapeutic potential [95,96].

Considering the increasing focus on antioxidant polyphenols, both as dietary components and valuable food industry by-products, our results hold notable relevance. Proper intercellular communication is fundamental for maintaining tissue integrity and preventing pathological processes, and therefore, our findings contribute to a better understanding of how resveratrol may support cellular functions through modulation of GJIC. However, it is important to note that the dose of resveratrol, its bioavailability, and long-term effects in humans remain under investigation, making it a promising, but not guaranteed, “miracle cure”.

## 5. Conclusions

In HeLa cells expressing exogenous Cx43 or Cx45, resveratrol induced time-, dose-, and connexin-dependent changes in GJ properties and cell viability. Resveratrol inhibited Cx43 GJ g_j_, but exerted a biphasic effect on Cx45 GJ g_j_, presumably due to differences in docking profiles, with a high-affinity docking site responsible for g_j_ stimulation absent in Cx43.

Resveratrol increased the connexin expression levels and enlarged GJ plaques in both Cx43- and Cx45-expressing cells.

Resveratrol provoked Cx43 phosphorylation, which was accompanied by the activation of the ERK1/2 signaling pathway, which, in turn, correlated with a reduction in HeLa cell viability and proliferation. Notably, the viability of HeLa cells expressing either of the connexins was more sensitive to resveratrol treatment compared with HeLa WT cells.

## Data Availability

The original contributions presented in this study are included in the article/Supplementary Materials. Further inquiries can be directed to the corresponding author.

## Supplementary Materials

The following supporting information can be downloaded at: https://www.mdpi.com/article/10.3390/antiox15010088/s1, Figure S1: Western blot replicates of total Cx43, Cx43-pS279, Cx43-pS282, Cx43-pS373, Cx43-pS368, ERK1/2, pERK1/2, and GAPDH for each set of experiment after 15 min of resveratrol treatment to HeLa Cx43 cells; Figure S2: Western blot replicates of total Cx43, Cx43-pS279, Cx43-pS282, Cx43-pS373, Cx43-pS368, ERK1/2, pERK1/2, and GAPDH for each set of experiment after 24 h of resveratrol treatment to HeLa Cx43 cells; Figure S3: Western blot replicates of total Cx45, ERK1/2, pERK1/2, and GAPDH for each set of experiment after 15 min and 24 h of resveratrol treatment to HeLa Cx45 cells; Figure S4: Western blot replicates of ERK1/2, pERK1/2, and GAPDH for each set of experiment after 15 min and 24 h of resveratrol treatment to HeLa WT cells; Figure S5: Typical images of F-actin and α-tubulin network in HeLa WT, HeLa Cx43, and HeLa Cx45 cells under control conditions and after the treatment with 100 μM of resveratrol for 24 h; Table S1: The most favorable amino acid residues for docking resveratrol in Cx43 and Cx45; Movie S1: Top docking sites of resveratrol to Cx43 protein. Movie S2: Top docking sites of resveratrol to Cx45 protein.

## References

[1] Rackauskas M., Neverauskas V., Skeberdis V.A. (2010). Diversity and properties of connexin gap junction channels. Medicina.

[2] Aasen T., Johnstone S., Vidal-Brime L., Lynn K.S., Koval M. (2018). Connexins: Synthesis, Post-Translational Modifications, and Trafficking in Health and Disease. Int. J. Mol. Sci..

[3] Söhl G., Willecke K. (2004). Gap junctions and the connexin protein family. Cardiovasc. Res..

[4] McNutt N.S., Weinstein R.S. (1970). The Ultrastructure of the Nexus: A Correlated Thin-Section and Freeze-Cleave Study. J. Cell Biol..

[5] Palacios-Prado N., Hoge G., Marandykina A., Rimkute L., Chapuis S., Paulauskas N., Skeberdis V.A., O’Brien J., Pereda A.E., Bennett M.V.L. (2013). Intracellular magnesium-dependent modulation of gap junction channels formed by neuronal connexin36. J. Neurosci..

[6] Bukauskas F.F., Verselis V.K. (2004). Gap junction channel gating. Biochim. Biophys. Acta.

[7] Dbouk H.A., Mroue R.M., El-Sabban M.E., Talhouk R.S. (2009). Connexins: A myriad of functions extending beyond assembly of gap junction channels. Cell Commun. Signal..

[8] Mickus R., Raškevičius V., Sarapinienė I., Mikalayeva V., Prekeris R., Skeberdis V.A. (2024). Phosphorylation-dependent allosteric regulation of Cx43 gap junction inhibitor potency. Biomed. Pharmacother..

[9] Zhang X.-F., Cui X. (2017). Connexin 43: Key roles in the skin. Biomed. Rep..

[10] Kopanic J.L., Al-mugotir M.H., Kieken F., Zach S., Trease A.J., Sorgen P.L. (2014). Characterization of the connexin45 carboxyl-terminal domain structure and interactions with molecular partners. Biophys. J..

[11] Desplantez T. (2017). Cardiac Cx43, Cx40 and Cx45 co-assembling: Involvement of connexins epitopes in formation of hemichannels and Gap junction channels. BMC Cell Biol..

[12] Wu J.-I., Wang L.-H. (2019). Emerging roles of gap junction proteins connexins in cancer metastasis, chemoresistance and clinical application. J. Biomed. Sci..

[13] Gleisner M.A., Navarrete M., Hofmann F., Salazar-Onfray F., Tittarelli A. (2017). Mind the Gaps in Tumor Immunity: Impact of Connexin-Mediated Intercellular Connections. Front. Immunol..

[14] Mickus R., Jančiukė G., Raškevičius V., Mikalayeva V., Matulytė I., Marksa M., Maciūnas K., Bernatonienė J., Skeberdis V.A. (2021). The effect of nutmeg essential oil constituents on Novikoff hepatoma cell viability and communication through Cx43 gap junctions. Biomed. Pharmacother..

[15] Leone A., Longo C., Gerardi C., Trosko J.E. (2019). Pro-Apoptotic Effect of Grape Seed Extract on MCF-7 Involves Transient Increase of Gap Junction Intercellular Communication and Cx43 Up-Regulation: A Mechanism of Chemoprevention. Int. J. Mol. Sci..

[16] Yan F., Tian X., Ma X. (2006). Effects of resveratrol on growth inhibition and gap-junctional intercellular communication of HepG2 cells. Nan Fang Yi Ke Da Xue Xue Bao.

[17] Nielsen M., Ruch R.J., Vang O. (2000). Resveratrol reverses tumor-promoter-induced inhibition of gap-junctional intercellular communication. Biochem. Biophys. Res. Commun..

[18] Martins L.A.M., Coelho B.P., Behr G., Pettenuzzo L.F., Souza I.C.C., Moreira J.C.F., Borojevic R., Radovan G., Carmem G., Fatima C.R. (2014). Resveratrol induces pro-oxidant effects and time-dependent resistance to cytotoxicity in activated hepatic stellate cells. Cell Biochem. Biophys..

[19] Han Y., Jo H., Cho J.H., Dhanasekaran D.N., Song Y.S. (2019). Resveratrol as a Tumor-Suppressive Nutraceutical Modulating Tumor Microenvironment and Malignant Behaviors of Cancer. Int. J. Mol. Sci..

[20] Chen Y., Li H., Zhang G., Wu Y., Xiao J., Liu J., Qiu P., Liu X., Sun L., Du B. (2020). Synergistic inhibitory effect of resveratrol and TK/GCV therapy on melanoma cells. J. Cancer Res. Clin. Oncol..

[21] Wang Y., Wang W., Wu X., Li C., Huang Y., Zhou H., Cui Y. (2020). Resveratrol Sensitizes Colorectal Cancer Cells to Cetuximab by Connexin 43 Upregulation-Induced Akt Inhibition. Front. Oncol..

[22] Bowers J.L., Tyulmenkov V.V., Jernigan S.C., Klinge C.M. (2000). Resveratrol acts as a mixed agonist/antagonist for estrogen receptors alpha and beta. Endocrinology.

[23] Truong V.-L., Jun M., Jeong W.-S. (2018). Role of resveratrol in regulation of cellular defense systems against oxidative stress. BioFactors.

[24] Dolinsky V.W., Chan A.Y.M., Robillard Frayne I., Light P.E., Des Rosiers C., Dyck J.R.B. (2009). Resveratrol Prevents the Prohypertrophic Effects of Oxidative Stress on LKB1. Circulation.

[25] Khan M.M., Ahmad A., Ishrat T., Khan M.B., Hoda M.d.N., Khuwaja G., Raza S.S., Khan A., Javed H., Vaibhav K. (2010). Resveratrol attenuates 6-hydroxydopamine-induced oxidative damage and dopamine depletion in rat model of Parkinson’s disease. Brain Res..

[26] Le H.T., Sin W.C., Lozinsky S., Bechberger J., Vega J.L., Guo X.Q., Saez J.C., Naus C.C. (2014). Gap Junction Intercellular Communication Mediated by Connexin43 in Astrocytes Is Essential for Their Resistance to Oxidative Stress. J. Biol. Chem..

[27] Hua R., Zhang J., Riquelme M.A., Jiang J.X. (2021). Connexin Gap Junctions and Hemichannels Link Oxidative Stress to Skeletal Physiology and Pathology. Curr. Osteoporos. Rep..

[28] Losso J.N., Truax R.E., Richard G. (2010). trans-resveratrol inhibits hyperglycemia-induced inflammation and connexin downregulation in retinal pigment epithelial cells. J. Agric. Food Chem..

[29] Cheng Y.-J., Chang M.-Y., Chang W.-W., Wang W.-K., Liu C.-F., Lin S.-T., Lee C.-H. (2015). Resveratrol Enhances Chemosensitivity in Mouse Melanoma Model Through Connexin 43 Upregulation. Environ. Toxicol..

[30] Kim J.H., Lee B.K., Lee K.W., Lee H.J. (2009). Resveratrol counteracts gallic acid-induced down-regulation of gap-junction intercellular communication. J. Nutr. Biochem..

[31] Balboa E., Saavedra F., Cea L.A., Ramírez V., Escamilla R., Vargas A.A., Regueira T., Saez J.C. (2020). Vitamin E Blocks Connexin Hemichannels and Prevents Deleterious Effects of Glucocorticoid Treatment on Skeletal Muscles. Int. J. Mol. Sci..

[32] Matusevičiūtė R., Ignatavičiūtė E., Mickus R., Bordel S., Skeberdis V.A., Raškevičius V. (2023). Evaluation of Cx43 Gap Junction Inhibitors Using a Quantitative Structure-Activity Relationship Model. Biomedicines.

[33] Lee H.-J., Cha H.J., Jeong H., Lee S.-N., Lee C.-W., Kim M., Yoo J., Woo J.-S. (2023). Conformational changes in the human Cx43/GJA1 gap junction channel visualized using cryo-EM. Nat. Commun..

[34] Goddard T.D., Huang C.C., Meng E.C., Pettersen E.F., Couch G.S., Morris J.H., Ferrin T.E. (2018). UCSF ChimeraX: Meeting modern challenges in visualization and analysis. Protein Sci..

[35] Wiederstein M., Sippl M.J. (2007). ProSA-web: Interactive web service for the recognition of errors in three-dimensional structures of proteins. Nucleic Acids Res..

[36] Laskowski R.A., MacArthur M.W., Thornton J.M. (2012). PROCHECK: Validation of protein-structure coordinates. International Tables for Crystallography.

[37] Jumper J., Evans R., Pritzel A., Green T., Figurnov M., Ronneberger O., Tunyasuvunakool K., Bates R., Zidek A., Potapenko A. (2021). Highly accurate protein structure prediction with AlphaFold. Nature.

[38] Kim S., Chen J., Cheng T., Gindulyte A., He J., He S., Li Q., Shoemaker B.A., Thiessen P.A., Yu B. (2019). PubChem 2019 update: Improved access to chemical data. Nucleic Acids Res..

[39] Koes D.R., Baumgartner M.P., Camacho C.J. (2013). Lessons learned in empirical scoring with smina from the CSAR 2011 benchmarking exercise. J. Chem. Inf. Model..

[40] Trott O., Olson A.J. (2010). AutoDock Vina: Improving the speed and accuracy of docking with a new scoring function, efficient optimization, and multithreading. J. Comput. Chem..

[41] Laskowski R.A., Swindells M.B. (2011). LigPlot+: Multiple ligand-protein interaction diagrams for drug discovery. J. Chem. Inf. Model..

[42] Skeberdis V.A., Rimkute L., Skeberdyte A., Paulauskas N., Bukauskas F.F. (2011). pH-dependent modulation of connexin-based gap junctional uncouplers. J. Physiol..

[43] Solan J.L., Lampe P.D. (2014). Specific Cx43 phosphorylation events regulate gap junction turnover in vivo. FEBS Lett..

[44] Lampe P.D., TenBroek E.M., Burt J.M., Kurata W.E., Johnson R.G., Lau A.F. (2000). Phosphorylation of connexin43 on serine368 by protein kinase C regulates gap junctional communication. J. Cell Biol..

[45] Pun R., Kim M.H., North B.J. (2023). Role of Connexin 43 phosphorylation on Serine-368 by PKC in cardiac function and disease. Front. Cardiovasc. Med..

[46] Liu F., Yang X., Geng M., Huang M. (2018). Targeting ERK, an Achilles’ Heel of the MAPK pathway, in cancer therapy. Acta Pharm. Sin. B.

[47] Gagliardi P.A., Pertz O. (2024). The mitogen-activated protein kinase network, wired to dynamically function at multiple scales. Curr. Opin. Cell Biol..

[48] Chang W.-S., Tsai C.-W., Yang J.-S., Hsu Y.-M., Shih L.-C., Chiu H.-Y., Bau D.-T., Tsai F.-J. (2021). Resveratrol inhibited the metastatic behaviors of cisplatin-resistant human oral cancer cells via phosphorylation of ERK/p-38 and suppression of MMP-2/9. J. Food Biochem..

[49] Yan H., Shao M., Lin X., Peng T., Chen C., Yang M., Zhong J., Yang J., Hui S. (2025). Resveratrol stimulates brown of white adipose via regulating ERK/DRP1-mediated mitochondrial fission and improves systemic glucose homeostasis. Endocrine.

[50] Chen X., Hu X., Li Y., Zhu C., Dong X., Zhang R., Ma J., Huang S., Chen L. (2019). Resveratrol inhibits Erk1/2-mediated adhesion of cancer cells via activating PP2A-PTEN signaling network. J. Cell. Physiol..

[51] Zhao Y., Tang H., Zeng X., Ye D., Liu J. (2018). Resveratrol inhibits proliferation, migration and invasion via Akt and ERK1/2 signaling pathways in renal cell carcinoma cells. Biomed. Pharmacother..

[52] Yu X., Sun Z., Nie S., Zhang T., Lu H. (2023). Effects of Resveratrol on Mouse B16 Melanoma Cell Proliferation through the SHCBP1-ERK1/2 Signaling Pathway. Molecules.

[53] Solan J.L., Lampe P.D. (2007). Key Connexin43 phosphorylation events regulate the gap junction life cycle. J. Membr. Biol..

[54] Roh D.S., Funderburgh J.L. (2011). Rapid changes in connexin-43 in response to genotoxic stress stabilize cell-cell communication in corneal endothelium. Investig. Ophthalmol. Vis. Sci..

[55] Laird D.W. (1996). The life cycle of a connexin: Gap junction formation, removal, and degradation. J. Bioenerg. Biomembr..

[56] Falk M.M., Kells R.M., Berthoud V.M. (2014). Degradation of connexins and gap junctions. FEBS Lett..

[57] Johnson A.M., Roach J.P., Hu A., Stamatovic S.M., Zochowski M.R., Keep R.F., Andjelkovic A.V. (2018). Connexin 43 gap junctions contribute to brain endothelial barrier hyperpermeability in familial cerebral cavernous malformations type III by modulating tight junction structure. FASEB J..

[58] Bukauskas F.F., Jordan K., Bukauskiene A., Bennett M.V., Lampe P.D., Laird D.W., Verselis V.K. (2000). Clustering of connexin 43-enhanced green fluorescent protein gap junction channels and functional coupling in living cells. Proc. Natl. Acad. Sci. USA.

[59] Palacios-Prado N., Briggs S.W., Skeberdis V.A., Pranevicius M., Bennett M.V.L., Bukauskas F.F. (2010). pH-dependent modulation of voltage gating in connexin45 homotypic and connexin45/connexin43 heterotypic gap junctions. Proc. Natl. Acad. Sci. USA.

[60] Palacios-Prado N., Sonntag S., Skeberdis V.A., Willecke K., Bukauskas F.F. (2009). Gating, permselectivity and pH-dependent modulation of channels formed by connexin57, a major connexin of horizontal cells in the mouse retina. J. Physiol..

[61] Marandykina A., Palacios-Prado N., Rimkutė L., Skeberdis V.A., Bukauskas F.F. (2013). Regulation of connexin36 gap junction channels by n-alkanols and arachidonic acid. J. Physiol..

[62] Pogoda K., Kameritsch P., Retamal M.A., Vega J.L. (2016). Regulation of gap junction channels and hemichannels by phosphorylation and redox changes: A revision. BMC Cell. Biol..

[63] Solan J.L., Lampe P.D. (2005). Connexin phosphorylation as a regulatory event linked to gap junction channel assembly. Biochim. Biophys. Acta.

[64] Shi Y., Hou X., Zhang X., Wang Y., Chen Y., Zou J. (2013). Inhibition of oxidized-phospholipid-induced vascular smooth muscle cell proliferation by resveratrol is associated with reducing Cx43 phosphorylation. J. Agric. Food Chem..

[65] Kim J.H., Choi S.H., Kim J., Lee B.K., Lee K.W., Lee H.J. (2009). Differential regulation of the hydrogen-peroxide-induced inhibition of gap-junction intercellular communication by resveratrol and butylated hydroxyanisole. Mutat. Res..

[66] Xie D., Zheng G.-Z., Xie P., Zhang Q.-H., Lin F.-X., Chang B., Hu Q.-X., Du S.-X., Li X.-D. (2017). Antitumor activity of resveratrol against human osteosarcoma cells: A key role of Cx43 and Wnt/β-catenin signaling pathway. Oncotarget.

[67] Aquilano K., Baldelli S., Rotilio G., Ciriolo M.R. (2009). trans-Resveratrol inhibits H_2_O_2_-induced adenocarcinoma gastric cells proliferation via inactivation of MEK1/2-ERK1/2-c-Jun signalling axis. Biochem. Pharmacol..

[68] Kameritsch P., Khandoga N., Pohl U., Pogoda K. (2013). Gap junctional communication promotes apoptosis in a connexin-type-dependent manner. Cell Death Dis..

[69] Ramos S., Alía M., Bravo L., Goya L. (2005). Comparative effects of food-derived polyphenols on the viability and apoptosis of a human hepatoma cell line (HepG2). J. Agric. Food Chem..

[70] Vizzotto M., Porter W., Byrne D., Cisneros-Zevallos L. (2014). Polyphenols of selected peach and plum genotypes reduce cell viability and inhibit proliferation of breast cancer cells while not affecting normal cells. Food Chem..

[71] Oprea D., Crisan D., Enache A. (2025). Polyphenolic Extracts From Green Vegetables as Promoters of Fibroblast Viability and Reducers of Oxidative Stress. Food Sci. Nutr..

[72] Du L., Chen E., Wu T., Ruan Y., Wu S. (2019). Resveratrol attenuates hydrogen peroxide-induced aging through upregulation of autophagy in human umbilical vein endothelial cells. Drug Des. Dev. Ther..

[73] Sahin E. (2025). Resveratrol suppresses cell viability and invasion in pancreatic cancer cells. Eskisehir Med. J. Eskisehir City Hosp..

[74] Gehm B.D., McAndrews J.M., Chien P.-Y., Jameson J.L. (1997). Resveratrol, a polyphenolic compound found in grapes and wine, is an agonist for the estrogen receptor. Proc. Natl. Acad. Sci. USA.

[75] Vargas-Villanueva J.R., Gutiérrez-Gutiérrez F., Garza-Ontiveros M., Nery-Flores S.D., Campos-Múzquiz L.G., Vazquez-Obregón D., Rodriguez-Herrera R., Palomo-Ligas L. (2023). Tubulin as a potential molecular target for resveratrol in *Giardia lamblia* trophozoites, in vitro and in silico approaches. Acta Trop..

[76] Schneider Y., Chabert P., Stutzmann J., Coelho D., Fougerousse A., Gossé F., Launay J.-F., Brouillard R., Raul F. (2003). Resveratrol analog (Z)-3,5,4′-trimethoxystilbene is a potent anti-mitotic drug inhibiting tubulin polymerization. Int. J. Cancer.

[77] Thomas E., Gopalakrishnan V., Hegde M., Kumar S., Karki S.S., Raghavan S.C., Choudhary B. (2016). A Novel Resveratrol Based Tubulin Inhibitor Induces Mitotic Arrest and Activates Apoptosis in Cancer Cells. Sci. Rep..

[78] Azios N.G., Krishnamoorthy L., Harris M., Cubano L.A., Cammer M., Dharmawardhane S.F. (2007). Estrogen and Resveratrol Regulate Rac and Cdc42 Signaling to the Actin Cytoskeleton of Metastatic Breast Cancer Cells. Neoplasia.

[79] Iwahara N., Azekami K., Hosoda R., Nojima I., Hisahara S., Kuno A. (2022). Activation of SIRT1 promotes membrane resealing via cortactin. Sci. Rep..

[80] Martin P.E.M., Evans W.H. (2004). Incorporation of connexins into plasma membranes and gap junctions. Cardiovasc. Res..

[81] Toyofuku T., Yabuki M., Otsu K., Kuzuya T., Hori M., Tada M. (1998). Direct association of the gap junction protein connexin-43 with ZO-1 in cardiac myocytes. J. Biol. Chem..

[82] Shaw R.M., Fay A.J., Puthenveedu M.A., von Zastrow M., Jan Y.-N., Jan L.Y. (2007). Microtubule Plus-End-Tracking Proteins Target Gap Junctions Directly from the Cell Interior to Adherens Junctions. Cell.

[83] Xiao D., Chen S., Shao Q., Chen J., Bijian K., Laird D.W., Alaoui-Jamali M.A. (2014). Dynamin 2 interacts with connexin 26 to regulate its degradation and function in gap junction formation. Int. J. Biochem. Cell Biol..

[84] Govindarajan R., Chakraborty S., Johnson K.E., Falk M.M., Wheelock M.J., Johnson K.R., Mehta P.P. (2010). Assembly of Connexin43 into Gap Junctions Is Regulated Differentially by E-Cadherin and N-Cadherin in Rat Liver Epithelial Cells. Mol. Biol. Cell.

[85] Silva G.A.L., Monte A.P.O., França A.T.V., Mota I.M., Oliveira Junior J.L., Andrade K.O., Souza L.M., Silva R.L.S., Guimarães V.S., Barberino R.S. (2024). Resveratrol attenuates doxorubicin-induced toxicity during in vitro culture of mouse-isolated preantral follicles. Zygote.

[86] Moreira-Pinto B., Costa L., Felgueira E., Fonseca B.M., Rebelo I. (2021). Low Doses of Resveratrol Protect Human Granulosa Cells from Induced-Oxidative Stress. Antioxidants.

[87] Salzano A., Albero G., Zullo G., Neglia G., Abdel-Wahab A., Bifulco G., Zicarelli L., Gasparrini B. (2014). Effect of resveratrol supplementation during culture on the quality and cryotolerance of bovine in vitro produced embryos. Anim. Reprod. Sci..

[88] Walle T., Hsieh F., DeLegge M.H., Oatis J.E., Walle U.K. (2004). High absorption but very low bioavailability of oral resveratrol in humans. Drug Metab. Dispos..

[89] Carrizzo A., Forte M., Damato A., Trimarco V., Salzano F., Bartolo M., Maciag A., Puca A.A., Vecchione C. (2013). Antioxidant effects of resveratrol in cardiovascular, cerebral and metabolic diseases. Food Chem. Toxicol..

[90] Walle T. (2011). Bioavailability of resveratrol. Ann. N. Y. Acad. Sci..

[91] Timmers S., Auwerx J., Schrauwen P. (2012). The journey of resveratrol from yeast to human. Aging.

[92] Patel K.R., Brown V.A., Jones D.J.L., Britton R.G., Hemingway D., Miller A.S., West K.P., Booth T.D., Perloff M., Crowell J.A. (2010). Clinical pharmacology of resveratrol and its metabolites in colorectal cancer patients. Cancer Res..

[93] Howells L.M., Berry D.P., Elliott P.J., Jacobson E.W., Hoffmann E., Hegarty B., Brown K., Steward W.P., Gescher A.J. (2011). Phase I randomized, double-blind pilot study of micronized resveratrol (SRT501) in patients with hepatic metastases--safety, pharmacokinetics, and pharmacodynamics. Cancer Prev. Res..

[94] Narayanan N.K., Nargi D., Randolph C., Narayanan B.A. (2009). Liposome encapsulation of curcumin and resveratrol in combination reduces prostate cancer incidence in PTEN knockout mice. Int. J. Cancer.

[95] Penalva R., Esparza I., Larrañeta E., Gonzalez-Navarro C.J., Gamazo C., Irache J.M. (2015). Zein-Based Nanoparticles Improve the Oral Bioavailability of Resveratrol and Its Anti-inflammatory Effects in a Mouse Model of Endotoxic Shock. J. Agric. Food Chem..

[96] Vries Kde Strydom M., Steenkamp V. (2021). A brief updated review of advances to enhance resveratrol’s bioavailability. Molecules.

