# Supplementary material for: Dose- and Time-Dependent Modulation of Cx43 and Cx45 Expression and Gap Junction Conductance by Resveratrol
Source: Antioxidants (Basel). 2026 Jan 9;15(1):88. doi: 10.3390/antiox15010088 (PMC12838047; doi:10.3390/antiox15010088)
Supplement: Supplementary file 1 [file antioxidants-15-00088-s001.zip › Supplementary Figure S5.pdf]

SUPPLEMENTARY FIGURE

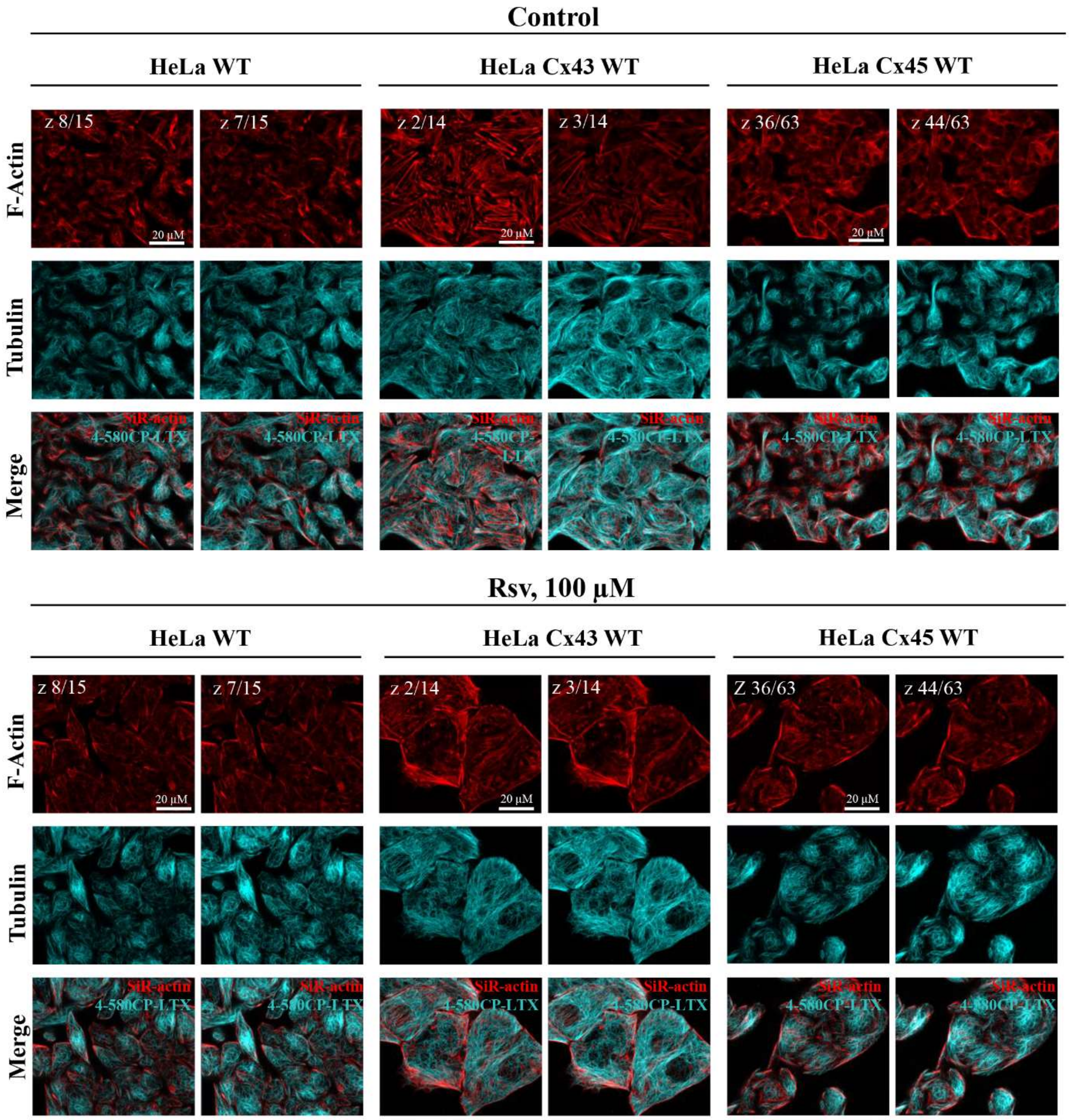

Figure S5. Typical images of F-actin and α-tubulin network in HeLa WT, HeLa Cx43, and HeLa Cx45 cells under control conditions and after the treatment with 100 µM of resveratrol for 24 h.
