# Supplementary material for: Dose- and Time-Dependent Modulation of Cx43 and Cx45 Expression and Gap Junction Conductance by Resveratrol
Source: Antioxidants (Basel). 2026 Jan 9;15(1):88. doi: 10.3390/antiox15010088 (PMC12838047; doi:10.3390/antiox15010088)
Supplement: Supplementary file 1 [file antioxidants-15-00088-s001.zip › Supplementary Figures S1-4.pdf]

SUPPLEMENTARY FIGURES

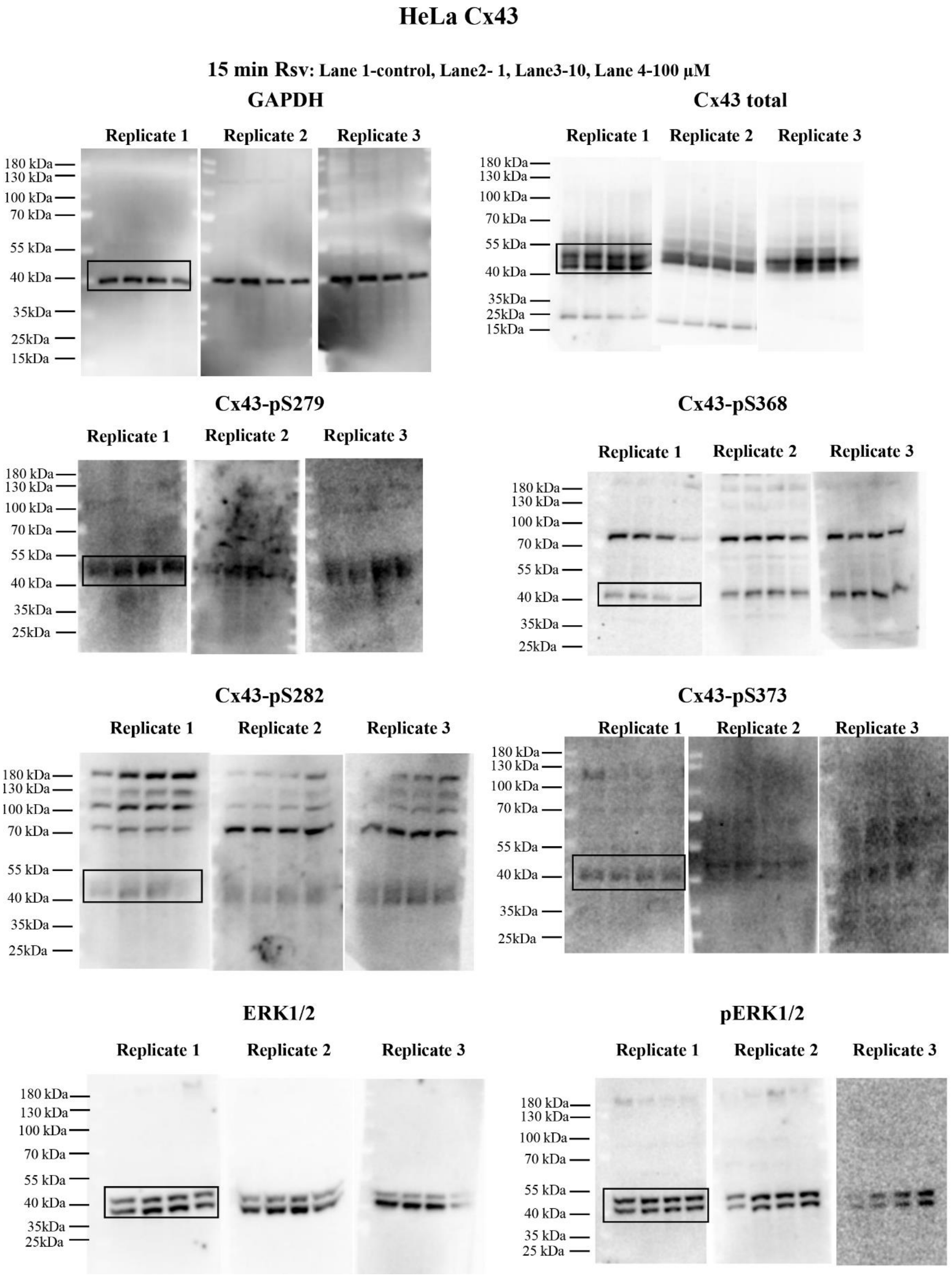

Figure S1. Western blot replicates of total Cx43, Cx43-pS279, Cx43-pS282, Cx43-pS373, Cx43-pS368, ERK1/2, pERK1/2, and GAPDH for each set of experiment after 15 min of resveratrol treatment to HeLa Cx43 cells.

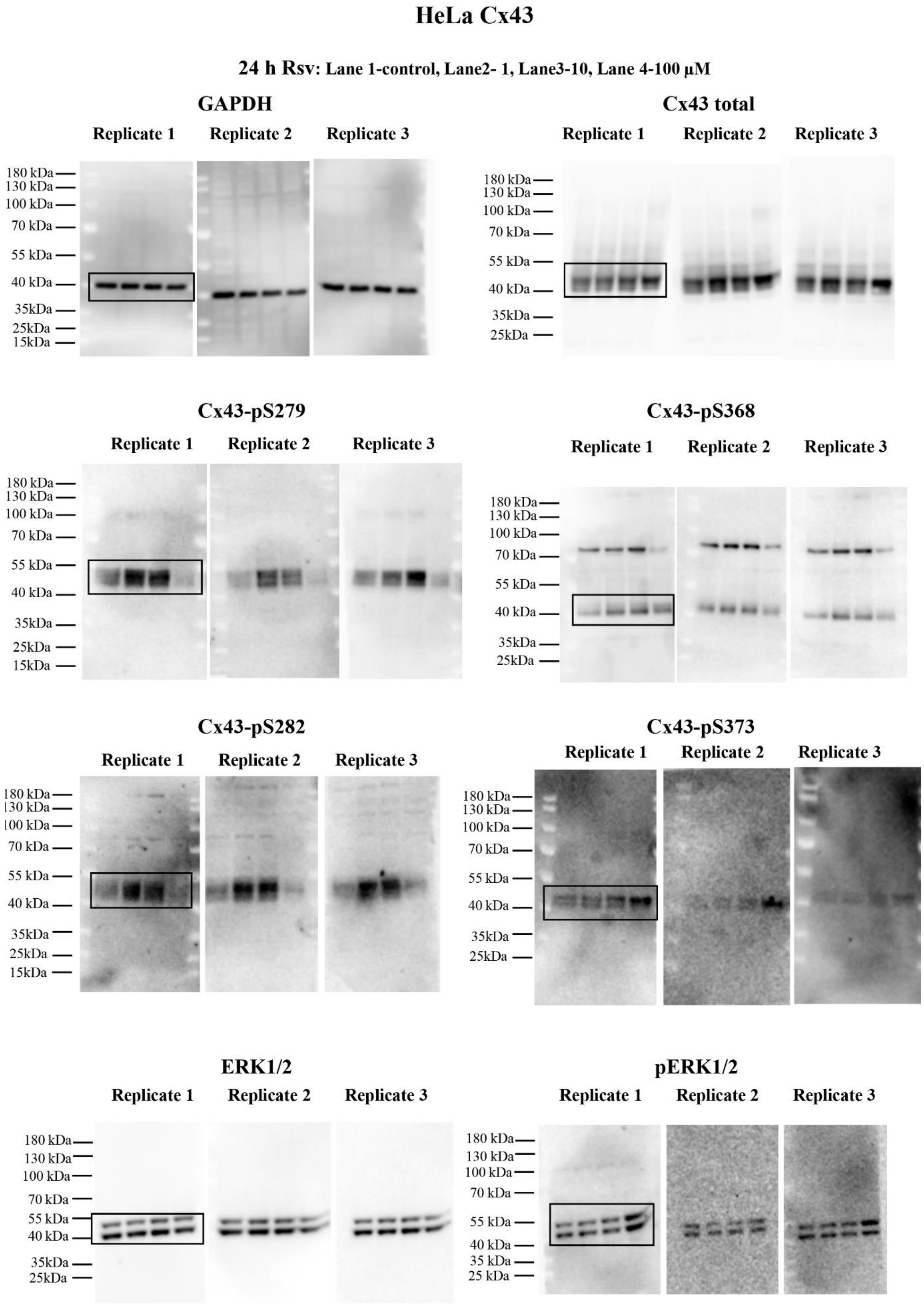

Figure S2. Western blot replicates of total Cx43, Cx43-pS279, Cx43-pS282, Cx43-pS373, Cx43-pS368, ERK1/2, pERK1/2, and GAPDH for each set of experiment after 24 h of resveratrol treatment to HeLa Cx43 cells.

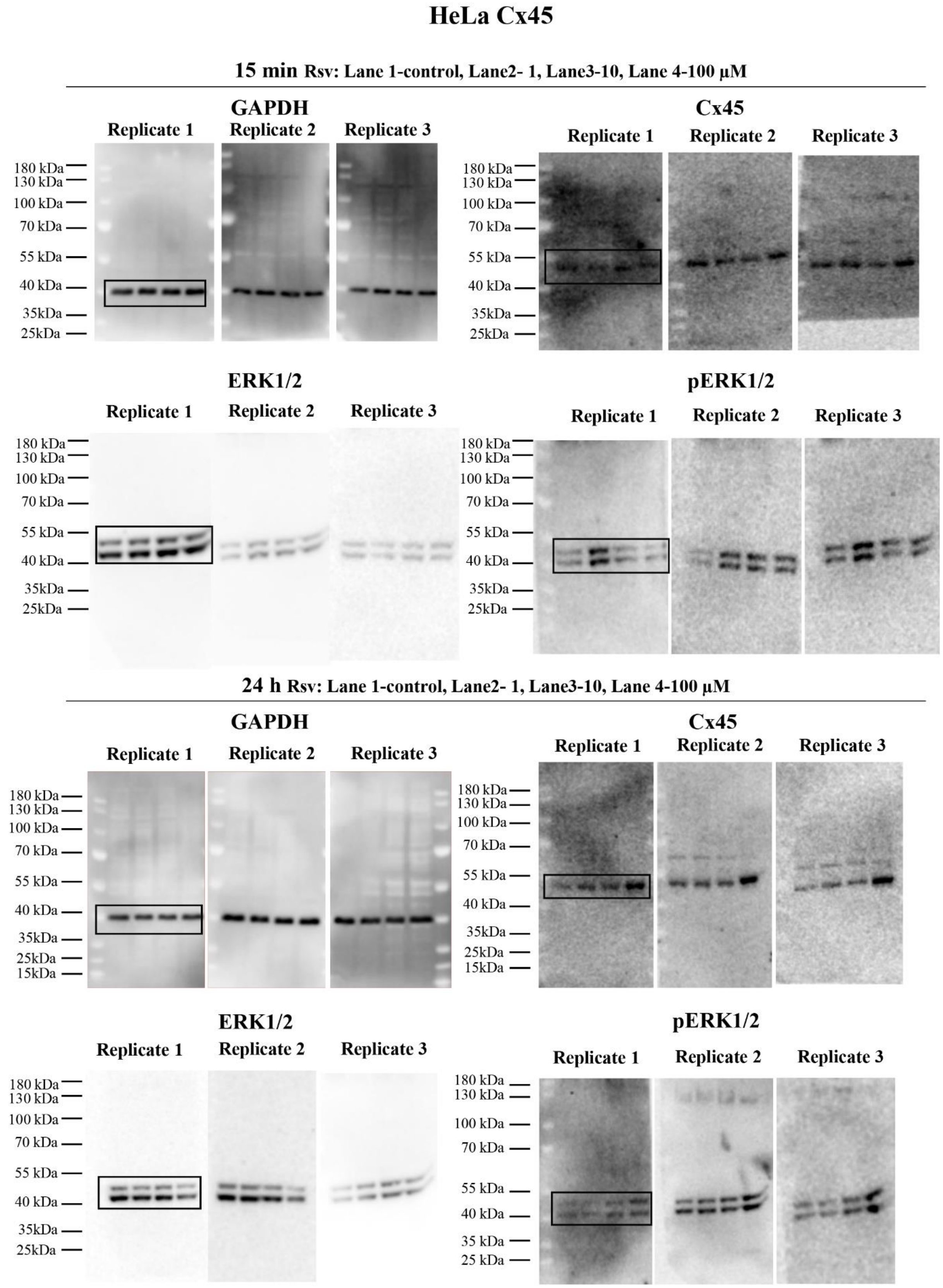

Figure S3. Western blot replicates of total Cx45, ERK1/2, pERK1/2, and GAPDH for each set of experiment after 15 min and 24 h of resveratrol treatment to HeLa Cx45 cells.

HeLa WT

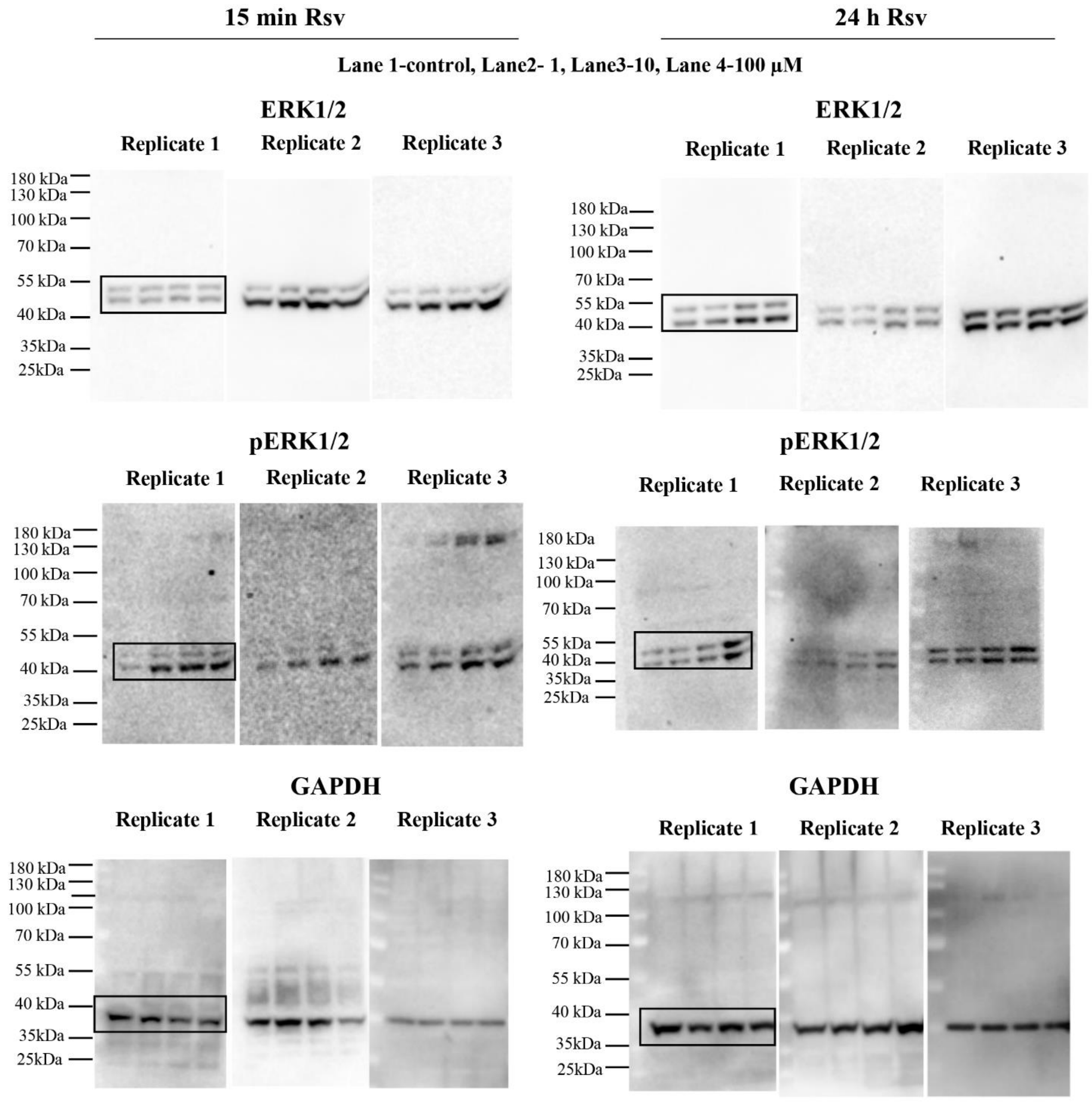

Figure S4. Western blot replicates of ERK1/2, pERK1/2, and GAPDH for each set of experiment after 15 min and 24 h of resveratrol treatment to HeLa WT cells.
