# Supplementary material for: Dose- and Time-Dependent Modulation of Cx43 and Cx45 Expression and Gap Junction Conductance by Resveratrol
Source: Antioxidants (Basel). 2026 Jan 9;15(1):88. doi: 10.3390/antiox15010088 (PMC12838047; doi:10.3390/antiox15010088)
Supplement: Supplementary file 1 [file antioxidants-15-00088-s001.zip › Supplementary Table S1.pdf]

SUPPLEMENTARY TABLE

Table S1. The most favorable amino acid residues for docking resveratrol in Cx43 and Cx45

**Docking sites**								
**1**	**2**	**3**	**4**	**5**	**6**	**7**	**8**	**9**
**Connexin 43**								
Ala168_1	Ala168_1	Leu91_1	Leu91_1	Ala168_1	Ala168_1	Ile172_1	Leu91_1	Phe32_2
Ile172_1	Ile172_1	Phe161_1	Phe161_1	Ile172_1	Ile172_1	Ile72_1	Phe165_1	Phe165_1
Phe165_1	Phe165_1	Phe165_1	Phe165_1	Phe165_1	Phe165_1	Leu80_1	Phe161_1	Phe169_1
Phe169_1	Phe169_1	Pro88_1	Pro88_1	Phe169_1	Phe169_1	Phe165_1	Pro88_1	Phe84_1
Phe84_1	Phe84_1	Val164_1	Val164_1	Phe84_1	Phe84_1	Phe169_1	Val164_1	Pro88_1
Val87_1	Val87_1	Val87_1	Val87_1			Phe77_1	Val87_1	Val87_1
						Phe84_1		
**Connexin 45**								
Ala85_1	Ala85_1	Ala85_1				Ala85_1		
**Ile82_1**	**Ile82_a**	**Ile82_1**	**Ile82_1**	**Ile82_1**		Ile82_1		Ile230_2
Leu28_2	Leu28_2	Leu28_2	Leu241_2	Leu241_2	Ile234_2	Leu28_2	Ile234_2	Ile234_2
			Leu28_2	Leu28_2	Leu71_1	Leu83_1	Leu71_1	Leu71_1
Leu83_1	Leu83_1	Leu83_1	Leu83_1	Leu83_1	Leu83_1		Leu83_1	Leu83_1
Leu9_1	Leu9_1	Leu9_1	Phe31_2	Phe31_2	Phe191_1	Phe31_2	Phe191_1	Phe191_1
Phe31_2	Phe31_2	Phe31_2						
Phe5_1	Phe5_1	Phe5_1	Phe5_1	Phe5_1	Phe31_2	Phe5_1	Phe31_2	Phe31_2
**Thr86_1**	**Thr86_1**	**Thr86_1**	**Thr86_1**	**Thr86_1**	Phe76_1	**Thr86_1**	Phe76_1	Phe76_1
			Trp24_2	Trp24_2	Phe79_1		Phe79_1	Phe79_1
**Val27_2**	**Val27_2**	**Val27_2**	**Val27_2**	**Val27_2**	Val238_2	**Val27_2**	Val238_2	Val238_2
Val30_2	Val30_2	Val30_2				Val30_2		
		Val34_2						
Val89_1	Val89_1	Val89_1						

In bold – amino acid residues for docking resveratrol in the niche of Cx45 TM1 and TM2.
